# Involuntary and voluntary memory retrieval relies on distinct neural representations and oscillatory processes

**DOI:** 10.1371/journal.pbio.3003258

**Published:** 2025-08-19

**Authors:** Malte Kobelt, Gerd T. Waldhauser, Marie-Christin Fellner, Nikolai Axmacher

**Affiliations:** Department of Neuropsychology, Faculty of Psychology, Institute of Cognitive Neuroscience, Ruhr University Bochum, Bochum, Germany; Universitat Jaume 1, SPAIN

## Abstract

Involuntary memory retrieval is a hallmark symptom of posttraumatic stress disorder and a frequent phenomenon in everyday autobiographical memory. However, the neural mechanisms that drive involuntary retrieval remain unclear. This study aims to elucidate how involuntary retrieval spontaneously initiates memory reactivation and how the reactivated neural representations differ in their content, distinctiveness and temporal compression from voluntary retrieval. Combining a visual half-field paradigm with electroencephalography recordings (EEG) in humans, we tracked reactivation of item-specific neural representations and sensory feature representations measured as representational similarity between different items sharing the same sensory feature – the visual field at encoding. We show that involuntary retrieval reactivated sensory feature-dependent yet item-unspecific representations via temporally extended memory replay, accompanied by rapid mid-frontal theta-power increases, indicating memory interference. This neural process differed from voluntary retrieval which recruited goal-directed memory search processes in prefrontal-medial temporal lobe theta-bands to reactivate temporally compressed item-specific representations devoid of visual field specific sensory feature representations at encoding. Our findings demonstrate that involuntary memories rely on distinct neural processes that access different representational formats compared to voluntary retrieval offering a nuanced understanding of episodic memory functioning relevant to psychological well-being.

## Abstract 2

Involuntary memory retrieval is a common phenomenon in autobiographical memory and a symptom of posttraumatic stress disorder. In an electroencephalography study, the authors elucidate the neural mechanism of involuntary memory, revealing that involuntary and voluntary memories rely on distinct neural processes.

## Introduction

In Marcel Proust’s famous novel “In Search of Lost Time”, the protagonist experiences the sudden recollection of long-forgotten events when tasting a particular combination of tea and bakery [1]. Phenomena of involuntary memory retrieval occur regularly in everyday life and may be as frequent as intentional, voluntary remembering [2–4]. Whereas involuntary retrieval in the healthy population typically involves emotionally positive or neutral memories [5], patients suffering from psychiatric disorders often experience the unwanted and uncontrollable intrusion of negative or traumatic experiences. Indeed, involuntarily intruding reminiscences of past events are among the most distressing symptoms of posttraumatic stress disorder (PTSD) [6,7] and are the focus of specialized psychotherapeutic interventions [8–10].

Despite their apparent ubiquity, importance for understanding psychological functioning, and relevance for mental health, involuntary memories have been studied only scarcely, especially when compared to the wealth of research on voluntary memory retrieval. Current theories on episodic memory often explicitly or implicitly refer to both voluntary and involuntary retrieval [11,12]. Contrarily, theories from clinical psychology propose that involuntary memories are stored in a dedicated, non-episodic memory system primarily processing perceptual information [7]. Indeed, phenomenological descriptions of involuntary memories by both healthy and mentally burdened individuals suggest that they contain several features which differ from voluntary memories: First, involuntary memories have been phenomenologically described as highly sensory in nature, despite sometimes considerable delays between encoding and retrieval [2,7]. By contrast, voluntarily retrieved memories have been suggested to primarily access the abstract “gist” of an experience [12–14] and typically lose sensory details during transformation processes following initial encoding, which involve further semanticisation and integration with other previous memories [15,16]. Second, involuntary memories are characterized by their generalized accessibility and tend to be easily triggered by ubiquitous perceptually similar cues even if those are encountered in a very different spatiotemporal context [6,17]. By contrast, voluntary memories typically maintain an episode-specific “gist” [18] reflected by the reinstatement of event-specific memory traces [19–21]. Third, detailed and extended descriptions of involuntary memories suggest that they consist in a temporally extended sequential “replay” of previous experiences [2]. By contrast, voluntary memory retrieval is typically compressed in time [22,23] and first accesses the gist of an event, which is then followed by a reconstruction of additional sensory information [11–13]. Fourth, involuntary memories occur rapidly and automatically when perceiving a cue, without the effortful and goal-directed control mechanisms associated with more challenging voluntary retrieval tasks [2,24,25]. Their intrusive occurrence may interfere with current cognitive goals and task demands [26,27]. Taken together, phenomenological descriptions suggest that involuntary and voluntary episodic memories differ substantially both with respect to the type of information they contain and regarding the cognitive processes involved.

Indeed, the few existing studies on the neurocognitive mechanisms of involuntary memory retrieval provide some evidence that they differ from the mechanisms that support voluntary retrieval [28]. Involuntary memories are characterized by a very rapid retrieval process, as indicated by a posterior event-related potential that occurs as early as 170 ms after cue onset [24]. In contrast to voluntary retrieval, involuntary retrieval lacks preceding or concurring top–down reconstruction attempts, as indicated by relatively less activity in memory control regions in the lateral prefrontal cortex [24,29,30]. However, despite these insights into the neurocognitive processes that differentiate involuntary from voluntary retrieval, previous studies were unable to track individual memories and compare their respective content depending on the type of retrieval. As a result, they could not account for the sensory detailedness, generalized accessibility, and protracted replay of involuntary memories.

**Fig 1:**
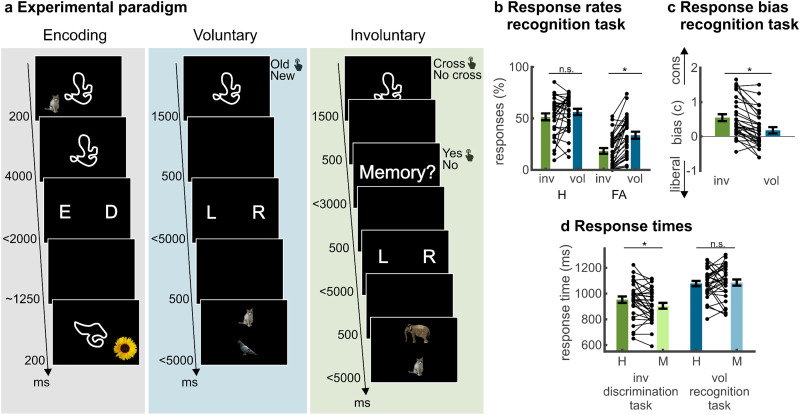
Memory characteristics and task-interference of involuntary retrieval. **A.** Experimental paradigm. Participants first encoded associations between abstract cues and everyday objects [53] that were presented in the left or right visual field, and decided whether the combination was easy (‘E’) or difficult (‘D’) to memorize. During voluntary retrieval, participants first conducted a recognition memory test of old and new abstract cues. Following a correct ‘old’ response, participants had to indicate the visual hemifield in which the object was encoded, followed by a forced choice test of object identity. In the involuntary retrieval test, participants were presented cues as well, but they were now instructed to indicate as fast and accurately as possible whether the abstract cues contained line crossings. Participants were then enquired about the automatic occurrence of an involuntary memory of the associated object. If a memory occurred, they again indicated the encoding hemifield and item identity. The experiment consisted of six encoding-retrieval blocks. **B.** Response rates for hits and false alarms during the voluntary recognition task and involuntary memory question following the visual discrimination task. **C.** Response bias for hits and false alarms during the voluntary recognition task and involuntary memory question following the visual discrimination task. **D.** Response times during visual discrimination in the involuntary retrieval task and recognition memory in the voluntary retrieval task. Hits and misses were defined based on the voluntary recognition task or involuntary memory question following the visual discrimination task. Error bars reflect standard errors of the mean. * *p* < .05; H, hits; FA, false alarms; M, misses; inv, involuntary; vol, voluntary; cons, conservative.

Here we directly compare neural representations of involuntary and voluntary memories of neutral visual material to investigate their basic neural processes independent of emotionality of stimulus material (Fig 1A). Natural images are encoded in several successive processing stages corresponding to different representational content, initially containing sensory features that may be shared among numerous episodes and eventually resulting in conceptual and semantic representations [31–33] with arguably fewer sensory features but higher specificity for a given episode. There is recent evidence that these different processing levels persist beyond encoding as “representational formats” of a memory trace that are flexibly accessible according to processing demands [14,34,35]. We hypothesized that voluntary retrieval of episode-defining gist information corresponds to compressed reinstatement of higher-order representational formats, essentially reversing the encoding cascade as shown by previous research [13,16]. Involuntary retrieval, in contrast, was expected to rely on temporally extended replay of visual-sensory formats that recapitulate the encoding process.

**Fig 2:**
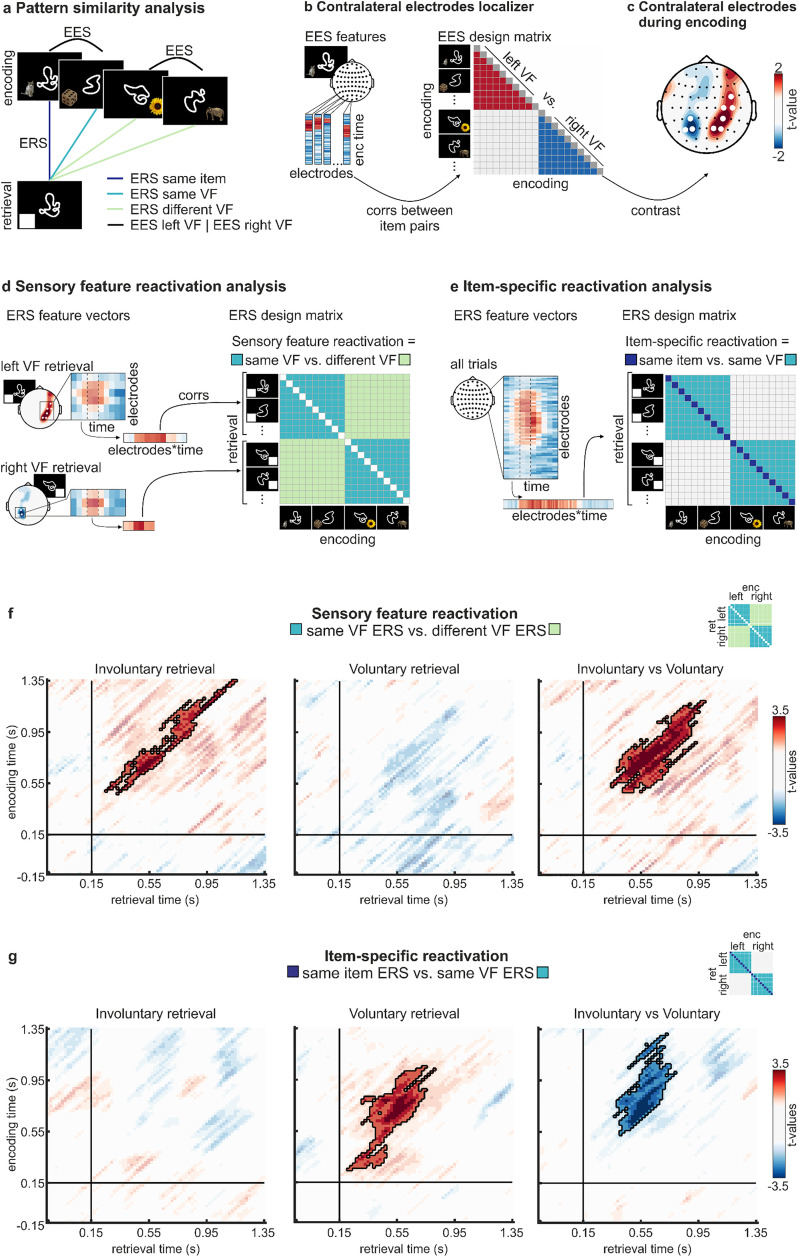
Involuntary retrieval reactivates sensory representations but lacks item-specific reactivation. **A.** Schematic depiction of encoding-retrieval similarity (ERS) and encoding-encoding similarity (EES) analyses. **B.** Contralateral electrodes localizer analysis at encoding. Left: EES feature vectors. EES analysis was based on the electrophysiological time courses of each electrode. Similarity between trials was calculated separately for each electrode across a selected encoding time period (50 to 950 ms after stimulus onset) resulting in an EES value between each item-pair at each electrode. Right: EES design matrix of localizer analysis. EES was contrasted between item-pairs presented in the left visual field vs. item-pairs presented in the right visual field. Positive clusters therefore represent electrodes most sensitive to information presented in the left visual field and negative clusters represent electrodes processing information presented in the right hemifield. **C.** Result of contralateral localizer analysis. Significant electrodes are highlighted as white bold dots. **D.** Sensory feature reactivation analysis. Left: ERS feature vectors. ERS was calculated across electrodes in the contralateral hemisphere most sensitive to lateralized visual processing. Using sliding time windows (illustrated as grey squares), we created electrode x time vectors which were used to calculate the similarity between encoding-retrieval pairs of each time window. Right: ERS design matrix. Sensory feature reactivation was calculated by comparing same VF ERS to different VF ERS. **E.** Item-specific reactivation analysis. Left: ERS feature vectors. ERS was calculated across all electrodes using the same sliding time window approach. Right: ERS design matrix. Item-specific reactivation was calculated as the difference between same item ERS and same VF ERS. **F.** Sensory feature reactivation analysis results. **G.** Item-specific reactivation analysis results. Left: Involuntary retrieval. Middle: Voluntary retrieval. Right: Interaction effects. Horizontal and vertical black lines indicate baseline time periods in each analysis (−0.15 s– 0.15 s). Baseline periods were not included into RSA permutation testing. EES – encoding-encoding similarity, ERS – encoding-retrieval similarity, VF – visual hemifield, enc – encoding, ret – retrieval. Reference images of the objects are adapted from [53].

In order to investigate how voluntary and involuntary memory retrieval reactivate representations that were established during encoding, we applied analyses of encoding-retrieval similarity (ERS) to EEG data (Fig 2A). Specifically, we correlated EEG signals during retrieval of individual items with EEG data during encoding. First, we tracked sensory feature reactivation. Specifically, we used a visual half-field paradigm that presents items in the left or right visual field during encoding which allowed us to identify memory representations of one specific visual feature – the hemifield at encoding – that represents retinotopic information and has been shown to be processed in early contralateral visual areas [36–41] and the intraparietal sulcus [41]. Accordingly, this visual field specific analysis was restricted to electrodes that were most sensitive to lateralized processing. This allowed us to track sensory representations referring to a specific visual feature. Second, we measured item-specific reactivation controlling for visual field specific sensory processing. In line with previous EEG studies tracking item-specific reactivation [42], this analysis was conducted across all electrodes because item identity and episodic gist has been suggested to be represented in widely distributed brain networks [43]. This approach tracked item-specific representations containing complex visuo-semantic information beyond the visual field in which an item had been presented. In a third step, we investigated whether voluntary and involuntary retrieval differed in terms of compression of encoded information, by contrasting the time courses of hemifield-specific and item-specific reactivation patterns between conditions. Finally, we applied time-frequency analyses to elucidate whether and how neural signatures of cognitive control and interference are involved during involuntary and voluntary retrieval. We hypothesized that voluntary retrieval induced delayed theta-power increases in the lateral prefrontal and medial temporal lobe which has been associated with strategic memory search [44–46] and recollection [47,48]. In contrast, involuntary retrieval was hypothesized to induce rapid midfrontal theta-power increases that have been related to cognitive interference [49] during memory retrieval [50], and to inhibition of unwanted memories during cognitive tasks [51].

Here we show that involuntary memory retrieval of neutral events reactivates information in different representational formats and recruits different cognitive processes compared to voluntary memory retrieval: Involuntary memory retrieval reactivates item-unspecific sensory feature representations over an extended time period, accompanied by rapid mid-frontal theta-power increases reflecting interference with ongoing cognitive tasks. By contrast, voluntary memory retrieval reactivates temporally compressed item-specific representations and recruits later theta-power increases in right lateral prefrontal cortex and left medial temporal lobe reflecting memory control processes.

## Results

### Behavioral results

Thirty-one participants first encoded associations between abstract line-drawings (in the center of a screen) and natural objects (presented in the left or right visual hemifield) [38]. During subsequent voluntary and involuntary memory periods, previously learned and novel abstract line drawings were presented on the screen. In the voluntary retrieval task, participants were explicitly instructed to remember the abstract cues, the associated object, and the object location. During involuntary retrieval, participants had to solve a visual discrimination task for each abstract cue and were instructed to report involuntarily occurring memories afterwards (Fig 1A). This memory question following the visual discrimination task was used to define hits or ‘false-alarm’ intrusions for the involuntary retrieval phase. During both voluntary and involuntary retrieval, participants performed a source memory and a subsequent forced-choice item memory task if they reported a memory.

We conducted behavioral analyses across all 31 participants to increase statistical power. However, this sample is not identical with the final EEG dataset, in which we excluded subjects due to handedness-issues (one subject), technical reasons (three subjects) or less than 10 fully remembered hits in the voluntary or involuntary retrieval condition (8 subjects), which was defined as a threshold to ensure a sufficient number of trials for EEG analysis (see S1 Text and S1 Fig for behavioral results in the EEG sample).

During both voluntary and involuntary retrieval, participants endorsed more previously seen items than new items as “old” (voluntary retrieval: hits (H) versus false alarms (FA): *t*_30_ = 9.177, *p* < .001, 95% CI = [0.18, 0.28], *d* = 1.65; involuntary retrieval: ‘hits’ versus ‘false’ alarm intrusions; *t*_30_ = 10.89, *p* <.001, 95% CI = [0.27, 0.39], *d* = 1.95; Fig 1B). Memory performance (*D’ *=* H*_z_* *− *FA*_z_) was higher during involun*t*ary than voluntary retrieval (involuntary retrieval: *M* = 1.15, *SD* = 0.61; voluntary retrieval: *M* = 0.67, *SD* = 0.44; *t*_30_ = 8.51, *p* < .001, 95% CI = [0.36, 0.59], *d* = 1.53). This was due to a lower FA ra*t*e in the involuntary condition (*t*_30_ = 7.80, *p* < .001, 95% CI = [0.11, 0.19], *d* = 1.40) which outweighed a trend for higher hi*t* rates in the voluntary condition (*t*_30_ = 1.75, *p* = .090, 95% CI = [−0.01, 0.10], *d* = 0.31; Fig 1C). A measure of response bias (**C* = −*0.5 * (*H*_*z *_+* FA*_*z*_)) confirmed more conservative responses in the involuntary (*M* = 0.55, *SD* = 0.56) *t*han in the voluntary (*M* = 0.18, *SD* = 0.52) condition (*t*_30_ = 5.29, *p* < .001, 95% CI = [0.23, 0.51], *d* = 0.95) [35]. This may reflect more effortful retrieval a*t*tempts during voluntary retrieval which at times lead to the inappropriate endorsement of new items as ‘old’, whereas ‘old’ responses for involuntary retrieval only occur under high certainty (see S2 Text for a more detailed discussion). Notably, differences in response bias, as observed in this study, have been shown to confound memory performance measure *D’* [52]. Accordingly, comparisons between involuntary and voluntary memory performances should be interpreted with caution.

We next assessed whether involuntary memory interfered with performance in the simultaneous visual discrimination task by comparing response times during successful and non-successful retrieval in the two conditions (Fig 1D). Notably, responses corresponded to recognition memory in the voluntary retrieval condition but perceptual ratings in the involuntary condition. A 2 × 2 ANOVA with retrieval condition (voluntary versus involuntary retrieval) and memory (hits versus misses) as factors revealed a significant main effect of retrieval condition (*F*_1,30_ = 52.75, *p* < .001, *η*_p_^2^ = 0.64), indicating shorter response times in the visual discrimination task as compared to voluntary retrieval. Moreover, we found a significant retrieval condition x memory interaction (*F*_1,30_ = 5.12, *p* = .031, *η*_p_^2^ = 0.15). While response times in the voluntary condition did not differ between hits and misses (*t*_30_ = 0.39, *p* = .703, 95% CI = [−33.63, 49.25], *d* = 0.07), *t*hey were longer when participants experienced involuntary memory intrusions as compared to when no intrusions were reported in the involuntary condition (*t*_30_ = 2.96, *p* = .006, 95% CI = [14.73, 80.39], *d* = 0.53). Thus, in line wi*t*h our hypotheses, involuntary memory retrieval created processing costs due to interference with the visual discrimination task.

We additionally compared reaction times during source and forced-choice item memory tasks between involuntary and voluntary retrieval as they may reflect differences in accessibility of memory content. Notably, we excluded six participants from the following analyses as they reported no misses in the forced-choice item memory task during involuntary memories. During voluntary retrieval, we found that response times were faster for hits compared to misses in source (*t*_23_ = −4.30, *p* < .001, 95% CI = [−146.27, −39.97], *d* = 0.88) and forced-choice item recognition tasks (*t*_23_ = −2.80, *p* = .010, 95% CI = [−128.25, −21.13], *d* = 0.57). While involun*t*ary retrieval also induced faster reaction times for hits compared to misses in the source memory task (*t*_23_ = −3.27, *p* = .004, 95% CI = [−94.78, −6.30], *d* = 0.67), *t*his effect did not reach significance in the forced-choice item recognition task (*t*_23_ = −0.53, *p* = .599, 95% CI = [−148.09, 54.22], *d* = 0.11). Memory effec*t*s in response times (hits versus misses) did not differ between involuntary and voluntary retrieval during source (*t*_23_ = 0.63, *p* = .532, 95% CI = [−16.18, 101.48], *d* = 0.13) or forced-choice i*t*em recognition tasks (*t*_23_ = 0.63, *p* = .538, 95% CI = [−86.65, 142.16], *d* = 0.13).

### Reinstatement of different representational formats during involuntary versus voluntary retrieval

We next assessed whether involuntary and voluntary retrieval reinstate memory traces with different representational formats, using complementary encoding-retrieval similarity (ERS) analyses [42,54]. ERS was quantified via Fisher z-transformed Spearman’s rank correlations between spatiotemporal time-domain EEG patterns during encoding and retrieval (Fig 2A). We restricted our analysis to trials in which both the identity of the cued object and its visual hemifield were correctly retrieved (“full-hits”) to control for potential differences in memory strength between involuntary and voluntary memories. Notably, we did not find any evidence for differences in memory strength between retrieval conditions as reflected by similar hit and full-hit rates between retrieval conditions in the EEG sample (Hits: *t*_18 _= −1.34, *p* = .198; full-hits: *t*_18_ = 1.79, *p* = .091).

First, we tested our hypothesis that involuntary but not voluntary retrieval relies on sensory feature reinstatement, focusing on electrodes that were most sensitive to lateralized sensory processing during encoding (using analyses of encoding-encoding similarity; Fig 2A–2C) [37,38]. We compared ERS of items presented in the same visual hemifield (same VF ERS) with items presented in different hemifields (different VF ERS; Fig 2D). To exclude item-specific effects, we selected only item-pairs referring to different objects during encoding and retrieval. We observed significant sensory feature reactivation during involuntary retrieval (Fig 2F). The observed cluster ranged from 250 ms to 1,150 ms during retrieval and from 470 ms to 1,350 ms during encoding (*t*_*sum*_ = 668.51, *p*_*corr*_ = .006, *d*_*lower*_* *= 0.61, *d*_*upper*_* *= 1.00). By contrast, we did not detect sensory feature reactivation during voluntary retrieval (*p*_*corr*_ > .935). Importantly, a direct comparison revealed more pronounced sensory feature reactivation during involuntary than voluntary retrieval in a cluster from 350 ms to 990 ms at retrieval and 490 ms to 1,190 ms at encoding (*t*_*sum*_ = 1172.50, *p*_*corr*_ < .001, *d*_*lower*_* *= 0.80, *d*_*upper*_* *= 1.15).

Next, we analyzed reactivation of item-specific information. In order to exclude effects of sensory feature reactivation of visual field information, this contrast controlled for the hemifield of each item, i.e., we compared ERS of same versus different items from the same hemifield (same item versus same VF ERS; Fig 2E). We first analyzed item-specific reactivation across all electrodes [42]. During involuntary retrieval, no significant ERS cluster was found (*p*_*corr*_ > .674; Fig 2G). In contrast, during voluntary retrieval, this analysis yielded a significant effect with a cluster ranging from 210 ms to 810 ms during retrieval and from 250 ms to 1,050 ms during encoding (*t*_sum_ = 963.92, *p*_corr_ = .018, *d*_*lower*_* *= 0.71, *d*_*upper*_* *= 0.87). Moreover, i*t*em-specific reactivation was higher during voluntary compared to involuntary retrieval (*t*_*sum*_ = −802.11, *p*_*corr*_ = .029, *d*_*lower*_* *= 0.69, *d*_*upper*_* *= 0.92) in a clus*t*er between 370 ms and 790 ms during retrieval and from 530 ms to 1,310 ms during encoding. Sensory or item-specific reactivation were not related to response times in source or forced-choice item recognition tasks (see S1 Table).

We then examined item-specific reactivation in two electrode subsets to determine whether it was predominantly observed in areas selective for lateralized sensory processing or in areas processing hemifield-independent information. For lateralized sensory processing electrodes, the same electrodes as in the sensory feature reactivation analysis were used, whereas areas processing hemifield-independent information were defined as the remaining electrodes, i.e., all those electrodes that did not exhibit lateralized sensory processing sensitivity during encoding.

In the lateralized sensory processing electrodes, no significant clusters of item-specific reactivation were observed during either involuntary (*t*_*sum*_ = 51.84, *p*_*corr*_ = .521) or voluntary retrieval (*t*_*sum*_ = 39.27, *p*_*corr*_ = .667). However, in hemifield-independent electrodes, item-specific reactivation occurred during voluntary retrieval (*t*_*sum*_ = 890.68, *p*_*corr*_ = .018, *d*_*lower*_* *= 0.69, *d*_*upper*_* *= 0.86), with a cluster spanning from 210 ms to 810 ms during retrieval and from 250 ms to 1,050 ms during encoding. No significant reactivation was observed in these electrodes during involuntary retrieval (*t*_*sum *_= 13.41, *p*_*corr*_ = .709). Consistently, voluntary retrieval showed higher item-specific reactivation than involuntary retrieval (*t*_*sum*_ = −841.29, *p*_*corr*_ = .042, *d*_*lower*_* *= 0.69, *d*_*upper*_* *= 0.88), with a cluster from 370 ms to 790 ms during retrieval and from 530 ms to 1,310 ms during encoding (see S3–S4 Texts and S2–S3 Figs for item-specific reactivation analysis in anatomically predefined regions or for a three-dimensional spatiotemporal cluster approach).

Overall, our results show that involuntary and voluntary retrieval reactivate information in different representational formats: Voluntary retrieval entails the reinstatement of item-specific activity patterns but no information about the sensory hemifield in which these items were encoded; reversely, involuntary retrieval reactivated sensory feature but not item-specific representations.

### Temporal dynamics and compression of memory reinstatement

Phenomenological reports described temporally extended replay of involuntary memories and compressed reinstatement of voluntarily retrieved episodes [2,7,55]. In line with these findings, temporal compression rates of group-level reactivation clusters differed between involuntary and voluntary retrieval in this study (see Fig 2F, left and 2G, center). We found that time points of the involuntary retrieval cluster contained a smaller number of encoding time points than time points of the voluntary retrieval cluster (involuntary retrieval: 5.5; voluntary retrieval: 12.03), indicating a compression rate that is half as large during involuntary compared to voluntary retrieval.

**Fig 3:**
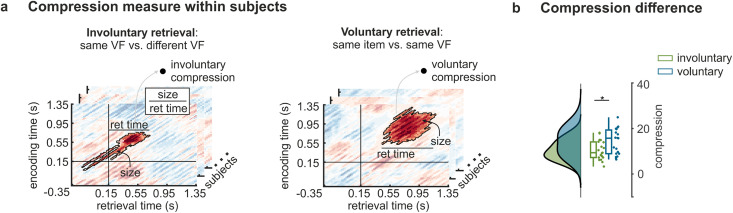
Temporally extended replay during involuntary retrieval. **A.** Illustration of analysis schema of the reactivation compression analysis. For each participant, we contrasted same VF ERS to different VF ERS during involuntary retrieval and same item ERS to same VF ERS during voluntary retrieval. Then, we selected the largest cluster for each contrast and calculated reactivation compression as the ratio of the whole cluster size (number of reactivated time points in the cluster) to the retrieval time. For illustration purposes, we present reactivation compression analysis in one representative participant. Horizontal and vertical black lines indicate baseline time periods in each analysis (−0.35 s–0.15 s). Note, cluster size and retrieval time were only calculated for data points within the time period of interest (0.15 s–1.35 s) **B.** Comparison of involuntary to voluntary reactivation compression using paired *t*-tests across subjects. Error bars reflect standard errors of the mean. * *p* < .05; enc, encoding; ret, retrieval.

We thus analyzed the temporal extension (or reversely, compression rates) in the two retrieval conditions by quantifying within-subject compression scores independent of previous group statistics (Fig 3C). For each participant, we first calculated sensory feature reactivation during involuntary retrieval (same VF versus different VF) and item-specific reactivation during voluntary retrieval (same item versus same VF) using paired *t*-tests across trials. We then identified a reactivation cluster in each participant which was defined as the largest cluster of all time points above a subject-specific threshold that was defined as the mean of all positive *t*-values. These subject-specific clusters were then used to calculate reactivation compression scores for each subject as the ratio between the number of all reactivated time points divided by the length of the retrieval time of the cluster (reactivation compression = cluster size/ cluster retrieval time) – i.e., corresponding to the average number of encoding time points reactivated at each retrieval time point. Using this approach, we again found that involuntary reactivation was less compressed than voluntary reactivation (*t*_18_ = 2.92; *p* = .009, 95% CI = [1.14, 6.53], *d* = 0.69; Fig 3D). Reactivation compression was not driven by the overall number of reinstated encoding time points (i.e., the sizes of reinstatement clusters), which did not differ between involuntary and voluntary retrieval (*t*_18_ = 0.79; *p* = .452, 95% CI = [−128.61, 303.77], *d* = 0.20). The result was also robust for various different subject-specific cluster thresholds (see S5 Text and S4 Fig). Furthermore, these results cannot be explained by differences in the variability of memory reactivation onsets between involuntary and voluntary memories (see S6 Text and S5 Fig). Together, these results show that involuntary retrieval does not only contain more pronounced sensory feature and less item-specific information, but also involves more temporally extended (less compressed) replay.

### Oscillatory signatures of involuntary and voluntary memory retrieval processes

The results presented thus far demonstrate that involuntary retrieval is characterized by distinct representational formats and reactivation dynamics that differ from those during voluntary retrieval. In our final analyses, we tested whether involuntary and voluntary retrieval also differed with regard to the recruitment of processes reflecting memory interference detection and goal-directed memory search. In other words, we tested which neurocognitive mechanisms, as reflected in oscillatory time-frequency patterns, drive involuntary versus voluntary memory retrieval. We compared activity during full-hits (see above) and correct rejections, i.e., novel abstract cues that did not elicit a memory. Since our hypotheses were specifically related to theta oscillations as a marker of both memory interference and goal-directed memory retrieval [47,56], we restricted our analysis to the theta frequency band (2–8 Hz) in a two-dimensional (time and space) cluster-based permutation statistic to correct for multiple comparisons [57,58].

**Fig 4:**
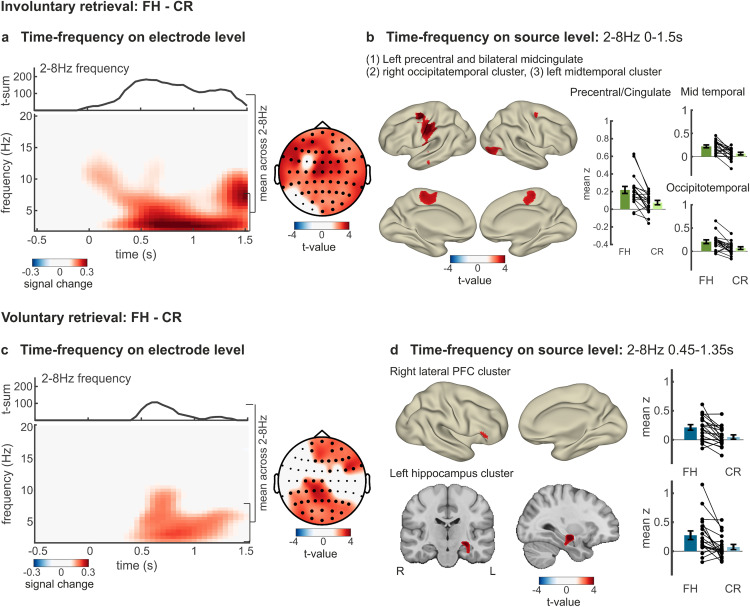
Distinct theta signatures of involuntary and voluntary memory retrieval. **A.** Left, top: time course of theta power in significant electrodes. T-sum values above zero indicate significant time points. Left, bottom: time-frequency plot of theta-band activity as mean across all significant electrodes. Time-frequency plots are only displayed for illustrative purposes and were not used for inference statistics. Right: topographical plot of significant electrode clusters. Significant electrodes are highlighted as bold dots. **B.** Source localization of significant theta-band cluster and bar plot depicting mean theta-power of full-hits (FH) and correct rejections (CR) within source localized clusters. **C.** Corresponding time-frequency analysis for voluntary retrieval. **D.** Source localization of significant theta-band clusters during voluntary retrieval and bar plots illustrating mean theta-power of full-hits and correct rejections within source localized clusters. Error bars depict standard errors of the mean.

For involuntary retrieval, this contrast revealed a significant theta power increase that emerged with cue onset and lasted until the end of the epoch with a peak at 550 ms (*t*_*sum*_ = 3415.48, *p*_*corr*_ < .001, *d*_*lower*_* *= 1.04, *d*_*upper*_* *= 1.14; Fig 4A, left) and a characteristic mid-frontal scalp distribution (Fig 4A, right). This theta-power increase was localized in left precentral gyrus and bilateral midcingulate gyrus and spread into the left superior temporal cortex according to source analyses (*t*_*sum*_ = 195.11, *p*_*corr*_ = 0.012; Fig 4B). Fur*t*hermore, we found two smaller clusters showing a trend in right occipitotemporal areas (*t*_*sum*_ = 35.84, *p*_*corr*_ = 0.051) and lef*t* mid temporal areas (*t*_*sum*_ = 31.67, *p*_*corr*_ = 0.054). We nex*t* tested this effect separately for slow- (2–4 Hz) and fast-theta (4–8 Hz) frequency bands revealing that theta-power increases during involuntary retrieval were observed for both slow- and fast-theta frequency bands (see S7 Text and S6 Fig). Additionally, we conducted control analyses to test whether theta-power differences reflect effects on actual theta oscillations or aperiodic slope. These analyses showed that involuntary retrieval was associated with both, theta oscillations (controlled for aperiodic slope) and a steeper aperiodic slope (see S8 Text and S7 Fig). These results are in line with previous proposals that midfrontal theta oscillations originating from the anterior cingulate cortex (ACC) signal detection of cognitive interference during several tasks including Stroop tasks [49] and memory interference [51].

For voluntary retrieval, the respective contrast revealed an increase of theta power as well but with a substantially later onset (450–1,350 ms after cue onset), a later peak (650 ms) and a right frontal and left parietal distribution (*t*_*sum*_ = 826.22, *p*_*corr* _= .012, *d*_*lower*_* *= 0.84, *d*_*upper*_* *= 0.98; Fig 4C). While increased theta-power in parietal regions was observed for slow- and fast-theta, theta-power increases in right frontal regions were predominantly related to slow-theta (see S7 Text and S6 Fig). Additionally, control analyses underlined that voluntary retrieval was related to differences in theta oscillations (controlled for aperiodic slope) in parietal and right frontal regions and a steeper aperiodic slope in left frontal regions that do not overlap with theta-related effects (see S8 Text and S7 Fig). Source analysis showed two prominent clusters located in right dorsolateral prefrontal cortex (*t*_*sum*_ = 63.50, *p*_*corr*_ = 0.025) and a trend in left medial temporal lobe with a peak in the hippocampus (*t*_*sum*_ = 19.70, *p*_*corr*_ = 0.068; Fig 4D). However, we acknowledge that precise localization to the hippocampus remains tentative due to the spatial resolution limitations of EEG signals. While the cluster in the medial temporal lobe was a trend and has to be interpreted with caution, both findings critically align with previous studies relating lateral PFC to goal-directed memory search and reconstruction via interactions with the medial temporal lobe [59], in particular via theta oscillations [25,47].

These results suggest that involuntary retrieval entails a rapid recruitment of processes related to sensory reactivation and interference, while voluntary retrieval relies on delayed recruitment of strategic memory search processes reactivating event-specific representations. However, a direct comparison of the difference between full-hits and correct rejections during involuntary versus voluntary memory retrieval revealed no significant difference in theta-power between both conditions (*p*_*corr*_ > .124).

## Discussion

Our results show that involuntary memory retrieval relies on substantially different behavioral characteristics, representational formats and oscillatory signatures compared to voluntary retrieval. These differences may account for four fundamental characteristics of involuntary memories: sensory-rich memory content, cue generalization, temporally extended memory replay, and enhanced requirements to resolve interference with ongoing cognitive tasks. We found that involuntary memory retrieval reinstates sensory feature representations via temporally extended replay and induced rapid midfrontal theta-power increases putatively reflecting memory interference. Contrarily, voluntary retrieval reactivates compressed item-specific representations, accompanied by theta-power increases in prefrontal areas and the medial temporal lobe probably reflecting memory control processes. These findings indicate that involuntary retrieval involves different neural processes and access different representational formats of a memory trace compared to voluntary retrieval.

Involuntary memories have long been suggested to occur due to rapid, uncontrolled reactivation of a memory trace after perceiving a cue [2,7]. However, empirical evidence that cue-driven reactivation is the essential process underlying involuntary retrieval has been scarce. Our results provide first evidence for this hypothesis as we show that involuntary memories rapidly reinstate sensory feature representations of an event after perceiving a memory cue. These findings are in line with classic theories that spontaneous, cue-driven reactivation is a fundamental retrieval process of episodic memory (called “ecphory”) [60] and consistent with a previous study which showed its causal role for memory retrieval [38]. This cue-driven reactivation differs substantially from typical voluntary retrieval processes which are believed to initiate a slower goal-directed memory search process primarily reinstating the gist of an event and then potentially reconstructing sensory details depending on task demands [18]. Notably, while involuntary memories have been proposed to rely exclusively on cue-driven reactivation, voluntary memories may rely on both retrieval processes depending on memory strength and task demands. If an item with high memory strength must be remembered in a voluntary retrieval task, cue-driven reactivation may be sufficient to retrieve the required information. This aligns with previous findings from our group of sensory reactivation during voluntary retrieval in an easier version of the visual half-field paradigm presenting only a single item instead of cue-target pairs, resulting in higher memory performance [38]. However, if sensory reactivation is not sufficient, goal-directed memory search enables voluntary retrieval to access additional relevant information especially during demanding memory tasks such as those in this study.

These different retrieval processes may provide the neural basis for the specific phenomenological characteristics of involuntary memories. First, our results suggest that reactivation of different representational formats accounts for the sensory nature of involuntary memories. While involuntary retrieval reinstated memory representations of one individual sensory feature in contralateral visual processing areas, we found that voluntary retrieval reactivated widely distributed item-specific representations, which may reflect the reinstatement of multiple mnemonic features across the ventral visual stream including sensory and higher-level semantic information going beyond the individual sensory feature of visual field information. This is in line with previous studies showing that voluntary retrieval particularly reactivates item-specific representations in the ventral visual stream and higher-order conceptual processing areas [19–21] and predominantly accesses conceptual information of an event [13,16] which may in particular comprise the gist of autobiographical experiences [18]. Hippocampal theta oscillations have been suggested to orchestrate this reactivation of item-specific representations [61] which is in line with increased theta oscillations in the medial temporal lobe during voluntary but not involuntary retrieval in our study. Notably, memory effects on reaction times – measured as reaction time differences of hits versus misses – did not differ between involuntary and voluntary retrieval, regardless of whether the task involved item identity or visual field location. Yet, previous studies underlined the more sensory nature of involuntary memories compared to effortful voluntary retrieval as involuntary memories induced clearer and more vivid mnemonic impressions [62]. Similarly, studies comparing effortless compared to effortful retrieval, closely aligned with the involuntary versus voluntary distinction, showed that effortless retrieval was predominantly experienced from a first-person, instead of a global third-person perspective [63]. The more pronounced reactivation of sensory feature representations during involuntary memories aligns with these findings and may contribute to their more sensory and vivid quality [2,7,64].

Second, the observed reactivation of item-unspecific sensory feature representations during involuntary memories may explain why perceptually similar memory cues can trigger involuntary memories (a phenomenon referred to as “cue generalization”) [17]. That is, involuntarily reactivated memory traces were not unique representations of a specific item, but instead were highly similar to representations of other items sharing similar sensory features (same hemifield at encoding). This result suggests that involuntary memories reactivated representations of such relatively broad sensory features instead of a comprehensive item-specific representation as observed during voluntary retrieval. Different items may be less distinguishable based on these features if they incorporate similar perceptual components such as the same hemifield in our study, or similar shapes or colors in real-life situations. For example, mnemonic discrimination between a parcel and a present may be difficult based on one individual sensory feature representation alone (e.g., both may be a cube) but would need to access additional conceptual information (“delivery” or “birthday”) to differentiate them. This finding further aligns with global matching principles suggesting that an increased overlap between information present at retrieval and encoding facilitates spontaneous memory retrieval [43]. It may also explain why memory cues resembling several sensory features of the original event – such as the combination of tea and bakery described by Proust – particularly trigger involuntary memories as we would expect them to result in overlapping sensory representations [2]. Notably, initial involuntary reactivation of sensory feature representations does not exclude reactivation of item-specific representations during later memory retrieval stages, which may explain why involuntary memories successfully accessed item identity information in our study.

Moreover, our finding that involuntary memories reactivate individual sensory feature representations bears similarity to previous studies on holistic memory retrieval. In these studies, participants learn multi-item scenes with systematic changes in item features [65–67]. Interestingly, they showed that holistic remembering and forgetting varies across memory levels as multi-item scenes were remembered more holistically than single items and their associated individual features [66]. Our results showed that involuntary memories reactivated individual sensory feature representations instead of item-specific representations, which may suggest less holistic memory compared to voluntary retrieval. On the other hand, one could also hypothesize that involuntary memories are more holistic than voluntary memories as they are more vivid, more specific and often induce the impression of reliving past experiences [2]. It would thus be an important field for future research to investigate whether involuntary memory is indeed more holistic than voluntary retrieval using different lure stimuli that differed from the previously encoded event in several distinct dimensions.

Finally, involuntary and voluntary reinstatement differed in the amount of memory compression, indicating temporally extended memory replay during involuntary memories. This is in line with exemplary reports in healthy participants and PTSD patients illustrating detailed and excessive descriptions of specific moments of the past during involuntary memories [2,7] which are particularly extended for fear-related material [55]. However, no previous study systematically investigated memory compression during involuntary retrieval. Highly compressed memories have been proposed as functionally advantageous because retrieval would take less time. This ability enables flexible transitions between memories and allows us to be more involved in the present moment [22]. Consistently, previous studies showed that voluntary retrieval reactivates compressed neural representations [68–70]. We expand these results by showing more extended memory replay during involuntary memory retrieval which occurs independent from personal goals or task demands inherent in voluntary retrieval. Notably, involuntary retrieval was less than half as compressed as voluntary retrieval. Our results point to two potential neurophysiological explanations why memory compression may differ between retrieval modes: First, involuntary retrieval involves less medial temporal lobe processing which is believed to facilitate the formation and retrieval of compressed memory representations [70,71]. Second, involuntary retrieval may take longer to complete memory reactivation as sensory representations involve larger neural populations, whereas conceptual representations engage a sparse neural code comprising only a limited number of finely tuned neurons [72–74]. According to this second argument, differences in memory compression may not only be due to different retrieval processes but also result from different representational formats of the retrieved contents. As a consequence of reduced medial temporal lobe involvement and predominant reactivation of sensory information, involuntary memories may unfold over longer time periods during which individuals are immersed in the past and distracted from ongoing task demands.

Involuntary memories induced different patterns of theta oscillations as compared to voluntary retrieval, supporting the seemingly spontaneous and uncontrolled occurrence of involuntary memories which are believed to interfere with ongoing cognitive tasks. While previous neuroimaging studies reported lower prefrontal activity and hence reduced goal-directed retrieval processes during involuntary memories [2,24,29,30], our results highlight the neural processes underlying involuntary memory interference by investigating theta-band oscillations. Delayed theta-power increases in lateral PFC and hippocampus are typically associated with strategic memory search [44–46] and recollection [47,48]. Contrarily, rapid theta-power increases over midfrontal electrodes are believed to result from detection of cognitive interference [56], in particular during competitive memory retrieval [49,50] and memory inhibition [51] which in turn initiates interference resolution. Congruently, voluntary retrieval was related to delayed theta-power increases (0.45 s–1.35 s after cue onset) in the lateral PFC and medial temporal lobe, whereas involuntary retrieval was characterized by rapid midfrontal theta-power increases localized in midcingulate and precentral gyri potentially reflecting memory interference processes. In line with this result, response times increased when involuntary memories occurred during the visual discrimination task. Interestingly, involuntary memories were associated with a steeper midfrontal aperiodic slope, suggesting a shift toward greater inhibitory tone due to changes in excitatory-inhibitory balance [75]. While speculative, this may further reflect conflict resolution processes, as suggested by prior studies [76,77]. Involuntary memories may thus have disrupted task-related cognitions, which lead to a detection of control demands in midcingulate cortex [78] and initiate processes of conflict resolution between different response options in left motor areas [56,79]. Notably, direct comparisons of theta-power changes during involuntary versus voluntary retrieval did not reveal significant differences between both retrieval phases.

Apart from their neural characteristics, our findings may help reconcile seemingly contradictory theories on involuntary memory retrieval. There is ongoing debate whether involuntary memories are stored in the same episodic memory system as voluntary memories or in a separate perceptual memory system [2,7]. This perceptual memory system was introduced by clinical theories to account for the more frequent occurrence of involuntary memories for traumatic compared to neutral experiences and their more sensory and vivid quality compared to voluntary memories [7]. Therefore, this perceptual memory system was assumed to primarily store traumatic memories due to excessive sensory processing during trauma exposure. Yet, cognitive theories question the existence of a second memory system in parallel to hippocampus-dependent episodic memory [2]. Our research brings a new perspective into this debate by introducing the concept of representational formats: We propose that involuntary and voluntary retrieval can access different aspects of a memory via the reactivation of different representational formats from a single episodic memory system.

Our study is consistent with existing frameworks which suggest that involuntary retrieval is not specific for traumatic memories but can also occur for neutral everyday events. In particular, the study of neutral memory content highlights that the involuntary reactivation of sensory representations likely evolved from one episodic memory system, rather than a distinct memory system exclusively storing traumatic content. Nevertheless, the devastating features of traumatic experiences at encoding may explain why they are particularly likely to result in involuntary memories. Specifically, excessive sensory processing during trauma exposure [80–82] might result in the formation of more pronounced sensory representations and has been previously shown to result in more intrusive memories of traumatic film footage [82]. While it is an open question whether traumatic memory intrusions indeed rely on similar neural processes as those observed in our study, our findings offer an overarching conceptual framework on involuntary memory that may in principle be applied to both everyday events and distressing and traumatic experiences. Yet, whether and how involuntarily reactivated sensory representations in our study are related to traumatic memory intrusions remains unclear, and future studies are needed to test whether and how they are affected by traumatic content and whether they may be altered in patients with PTSD.

While this study was designed to investigate whether sensory feature reactivation is a basic neural process during involuntary memories, it has several limitations. First, this study applied a visual half-field paradigm to track a specific sensory feature during involuntary retrieval. At this point, it thus remains unclear whether this finding generalizes to other sensory features or whether it is specific to retinotopic visual hemifield information. Second, while we were able to detect item-specific reactivation and theta-power increases in the medial temporal lobe during voluntary retrieval, the low spatial resolution of scalp EEG limited identification of the precise brain areas involved in item-specific reactivation; our study also cannot unequivocally state that theta-power increases indeed originated from the hippocampus. Intracranial EEG studies using this task would help understand differences between the neural basis of voluntary and involuntary retrieval in more detail. Third, previous work emphasized the role of specific frequency bands for memory reactivation including theta, alpha/beta and gamma frequency bands [83–85]. Here, we applied time-domain RSA to track memory reactivation. While this is an established method to track memory reactivation [42], this approach limits inferences on its underlying spectral sources. However, these are important questions that are beyond the scope of our study and should be addressed by future work applying frequency-resolved representational similarity analyses or phase-amplitude coupling. Finally, the results of this study refer to neutral memories in healthy participants and therefore need to be translated into a clinical setting to test whether and how sensory feature reactivation is altered by negative events and psychiatric patients.

Moreover, one may argue that the observed memory reinstatement effects during involuntary retrieval could be contaminated by the sequence and the experimental design of the involuntary and voluntary retrieval tasks. Specifically, since the involuntary phase was always preceded by the voluntary task and since the involuntary retrieval phase also involved a question whether a memory intrusion occurred during the visual discrimination task, participants may have engaged in voluntary retrieval of prior events also in the involuntary task. From a different angle, the involuntary task may have been contaminated by the effects of the dual-task situation, created by demanding participants to both focus on visual discrimination and report intrusions. Thus, differences between both tasks may not reflect distinctions between involuntary and voluntary retrieval but instead arise from retrieval practice, due to the fixed task order, or retrieval inhibition. However, neither of these explanations can account for the sensory feature reinstatement effects we observed during involuntary retrieval for the following reasons. First, if anything, repeated encoding may promote semanticisation of a memory trace instead of fostering sensory feature reinstatement. For example, previous studies showed that repeated encoding strengthens representations of higher-level semantic features of an event [14,86]. Similarly, another study indicated that repeated encoding and retrieval practice promotes memory performance for semantic rather than visual features [16]. Thus, if retrieval practice increases semanticisation, it cannot account for our finding of the reactivation of sensory feature representations during involuntary memories. Notably, it could be argued that it is this semanticisation that leads to a reduction in item-specific reactivation during involuntary memories, especially if one considers item-specific reactivation a more sensitive marker of sensory representations than sensory feature reactivation. Yet, while some studies have linked item-specific reactivation to occipital areas [87], most research has emphasized the role of the anterior ventral visual stream and medial temporal lobe for item-specific reactivation [19,88–92] in line with the view that they consist of a complex combination of visuo-conceptual information necessary to distinguish individual items. This also aligns with our finding that item-specific reactivation occurred across all electrodes and was primarily located in visual field independent processing regions. Thus, we consider it unlikely that item-specific reactivation in our study more reliably captured sensory representations than sensory feature reactivation. Second, it could be argued that the involuntary retrieval task’s dual-task design might have increased cognitive demands, leading to retrieval inhibition. In this scenario, participants may have initially retrieved memories that they then needed to suppress in order to focus on the visual discrimination task, potentially disrupting reactivation. However, disrupted retrieval processes are again unlikely to explain why sensory reactivation was observed during the involuntary retrieval task but not during voluntary retrieval. Instead, this dual-task effect may account for the absence of item-specific reactivation, which may have been reactivated following cue-driven sensory reactivation but was inhibited due to task demands. While future studies are necessary to completely rule out these potential limitations, we believe both factors are unlikely to account for the sensory reinstatement effects during involuntary retrieval.

In conclusion, our study sheds new light on the underexplored phenomenon of involuntary memories relevant for understanding both psychological functioning and mental health. Our results elucidate a distinct neural process involved in involuntary memory retrieval which accesses different neural representations from episodic memory compared to voluntary retrieval. These findings deepen our understanding of memory retrieval processes, providing insights into the multifaceted nature of human memory traces.

## Materials and methods

### Participants

Thirty-one right-handed subjects (17 female) participated in the present study with a mean age of M = 23.87 (SD = 4.86), ranging from 18 to 37 years. Twelve subjects had to be excluded from the EEG analysis (*n* = 19, age: *M* = 24, *SD* = 4.55, 10 females) due to handedness-issues (one subject), technical reasons (three subjects) or less than 10 fully remembered hits in the voluntary or involuntary retrieval condition (8 subjects), which was defined as a threshold to ensure a sufficient number of trials for EEG analysis [42]. Sample size was determined based on previous EEG and fMRI studies from our lab and other research groups using similar stimuli and designs. These previous studies showed that a sample size of approximately 20 participants allows one to reliably measure sensory processing using visual half field paradigms [37–39,41,93] and to track item-specific reactivation using the same RSA approach on EEG data as in the current study [42]. The experimental procedure and methods adhered to the Declaration of Helsinki and were approved by the local ethics committee at the Faculty of Psychology, Ruhr University Bochum (approval no. 266). All participants provided written informed consent.

### Stimulus material

The stimulus set comprised 288 randomly matched item pairs each consisting of an abstract line drawing [94] and a picture of an everyday object. Using a previously published controlled stimulus set [53], item pairs were segmented into six sets, for which the everyday objects were balanced regarding visual complexity and object category. During each block, 16 item pairs were shown during encoding. These item pairs were shown again during both voluntary and involuntary retrieval, randomly intermixed with 16 new pairs during each of the two retrieval conditions (same number of old and new items during each retrieval block). To ensure detailed memory retrieval during the forced-choice recognition task, a new object from the same category as the old object was shown as a distractor.

### Procedure

The experiment comprised three phases which were repeated in six blocks (Fig 1A). The order of the phases was fixed starting with encoding, followed by voluntary retrieval and ending with involuntary retrieval. The stimuli in each block were presented in random order. Answers were given by a button press with the right hand using the right index and middle finger. During the encoding phase, participants were asked to learn 16 cue-object pairs. At the beginning of each trial, participants had to focus on a fixation cross (duration randomly jittered between 1,000 and 1,500 ms). Afterwards, a cue-object pair was presented consisting of an abstract line drawing in the center of the screen (cue; 4,000 ms) and a picture of an everyday object in the left or right hemifield (object; 200 ms). The short presentation of the object prevented saccades to the laterally presented target and therefore ensured contralateral processing of visual information. Following stimulus presentation, subjects were instructed to rate the learning difficulty of the cue-object pair to strengthen stimulus processing. To prevent memory rehearsal, subjects counted down from a random number between 100 and 999 in steps of three for 16 s after the encoding phase.

In the voluntary retrieval phase, each trial started with an old/new recognition test in which one of the 16 previously presented abstract cues or a new line drawing was shown in the center of the screen (1,500 ms). If participants indicated to remember the cue, they were asked to indicate in which visual hemifield the associated target was displayed during encoding (<5,000 ms), followed by a forced-choice recognition task asking to identify the associated target from two simultaneously displayed objects (<3,000 ms). If participants gave an ‘old’ response to a new item, they received feedback after the source memory task that the line drawing was new. If no memory was reported although an old cue was shown, the associated object was presented in the same visual hemifield again. This was done to increase the number of subsequent involuntary memories that may otherwise result in only few involuntarily retrieved memories, and given the general low memory performance for abstract line drawings [94].

The involuntary retrieval phase was designed to be as similar as possible to the voluntary task. We again presented the 16 previously learned and 16 new abstract line drawings in the center of the screen for 1,500 ms, but now instructed participants to indicate as fast as possible whether the lines in the cues crossed (e.g., as in the number “8”) or not (as in the number “0”). Thus, participants had to focus on the visual appearance of previously learned cues – a task that was designed to facilitate the induction of involuntary memories. The instructions emphasized that accuracy and reaction time on the visual discrimination task was of highest importance since we aimed to test the effect of intrusive involuntary memories on this task. To underline task relevance, participants received visual feedback if they responded too slow (<1,500 ms) or incorrectly. After each trial, participants were asked to indicate if they had incidentally experienced any involuntary memories (<3,000 ms) which may refer to the memory cue, item location, or the presented item. If they reported a memory, they were asked to respond to the source memory task and the forced-choice recognition task referring to the previously presented object as in the voluntary retrieval phase.

### EEG acquisition and measurement

EEGs were recorded with an elastic cap consisting of 64 Ag/AgCl skull electrodes placed according to the international 10–20 recording system and with a sampling rate of 500 Hz (BrainCap MR; Brain Products, Gilching, Germany), referenced to electrode FCz during recording and offline-referenced to common average. Impedances were kept below 10 kΩ. To correct for eye blinks and artefacts, trials were first excluded by detailed visual exploration. Furthermore, all epochs containing artifacts related to vertical eye movements as identified by careful visual inspection were excluded from further analyses. Afterwards, independent component analysis (ICA) was used to correct remaining artefacts. Preprocessing, time-frequency and source analyses relied on the fieldtrip toolbox and custom MATLAB scripts (The Mathworks, Munich, Germany). For ERS, we employed a self-written analysis pipeline in MATLAB. Only fully remembered trials, i.e., hits with correct source and item memory, were included for the EEG analysis.

### Statistical analysis of the behavioral data

All statistical analyses of the behavioral data were conducted for the whole sample, including participants who were later excluded from EEG analysis. First, recognition performance was analyzed using the discrimination index *D’* by subtracting the normalized false alarm rate (new items incorrectly labeled as old) from the normalized hit rate (correctly recognized old items) [95]. A *D’* value of zero indicates memory performance on chance level.

We also examined *Criterion C* as a measure of response bias [95], which describes the tendency to report a memory under a state of uncertainty. A *C* value above 0 implies that participants tended to not report a memory if they were uncertain and a value below 0 indicates that memories tended to report memories in states of uncertainty. Thus, *C* values above 0 indicate a conservative response bias and *C* values below 0 indicate a liberal response bias. *C* is calculated as **C* = −*0.5*(*FAR*_*z*_ + *HR*_*z*_*).* Paired *t*-tests were used to examine the difference between involuntary and voluntary retrieval and Cohen’s *d* was calculated to report effect sizes.

Additionally, we analyzed the main effects of retrieval phase and object memory and their interaction on mean response times using a 2 (phase: voluntary versus involuntary) × 2 (memory: hits versus misses) repeated measures ANOVA. Additionally, effect sizes were estimated using eta-squared. Post-hoc two-sample *t*-tests comparing fully remembered and missed targets during involuntary or voluntary retrieval unraveled the direction of the interaction effect.

### ERS—neural pattern similarity during involuntary and voluntary retrieval

#### Analysis schema.

ERS of the EEG signals enables investigating the temporal dynamics of memory reinstatement [42]. We expected reinstatement of sensory feature information during involuntary retrieval, and reinstatement of item-specific information during voluntary retrieval. ERS was applied by correlating the temporally resolved neural patterns of encoding trials with those of retrieval trials yielding a measure of reinstatement. To analyze the reinstatement of sensory feature and item-specific activity, we conducted two RSA procedures. The sensory analysis calculated the contrast of ERS of different objects presented in the same visual field with ERS of different objects presented in different visual fields (Fig 2A). The item-specific analysis compared ERS of the same objects presented in the same visual field to ERS of different objects in the same visual field (Fig 2A).

#### Encoding-retrieval similarity.

Before implementing the ERS analysis, we first *z*-transformed the artifact-corrected EEG data over electrodes and sampling points including all trials and down-sampled the EEG to 50 Hz [42]. We selected only encoding and retrieval trials of later fully remembered objects to control for memory strength between retrieval conditions. Moreover, fully remembered trials reflect successful memory retrieval processes whereas the cognitive processes underlying incomplete hits or misses are less clear, challenging interpretation of results. Overall, we included on average 25 full-hit trials during involuntary memory retrieval per participant (SD = 10.91; range = 10–43) and 20.68 full-hit trials during voluntary memory retrieval (SD = 8.29, range = 11–43). For computation of temporal pattern similarity, the *z*-transformed EEG data were segmented into overlapping time windows of 300 ms with increments of 20 ms. For each time window, the resulting matrix of 16 time points x 64 electrodes was concatenated, leading to a one-dimensional vector with combined spatial and temporal information. All time × channel vectors during encoding were then correlated with all time × channel vectors during the retrieval period using Spearman’s correlations and were then Fisher-*z*-transformed. This results in ERS matrices for all combinations of encoding time bins and retrieval time bins. For statistical testing, a cluster-based permutation approach was again used to correct for multiple comparisons while taking different trial numbers in the different conditions into account, although the number of full-hits included into EEG analyses did not differ between retrieval phases (*t*_18_ = 2.03, *p* = .058). To this end, the trial labels were shuffled in each subject for 1,000 times generating random trial sets reflecting the number of trials in the original conditions. In a second step, the two conditions of interest were contrasted for each random permutation data set, clusters of significant adjacent encoding/retrieval time bins (threshold *p* < 0.05) were identified, and the sum of *t*-values in each cluster was calculated. We then selected the cluster with the highest positive sum of *t*-values and lowest negative sum of *t*-values of each permutation to construct null distributions of effects under the existing bias in trial number. If no *t*-value reached significance in a given permutation, a cluster value of 0 was assigned. Finally, we contrasted the two conditions of interest in the original data and assessed significance of the empirical clusters by calculating the rank of the cluster *t*-values in the distribution of random data. A cluster was interpreted as significant if a higher or lower cluster *t*-value was found in less than 2.5% of the random permutations [42].

#### Contralateral electrode localizer analysis.

To investigate reinstatement of sensory-level information, we conducted ERS analyses on the reinstatement of lateralized EEG activity of objects presented in individual hemifields (see Fig 2A) [37,38]. Before extracting ERS values, we identified those electrodes that were most sensitive to sensory processing during encoding. First, we conducted a spatiotemporal pattern similarity analysis as described above, but only for encoding trials, to reveal the time-window of sensory-specific activity (encoding-encoding similarity, EES). Assuming that sensory-specific activity is highly dependent on the hemifield of presentation, we contrasted pattern similarity of encoding trials in which items were presented in the same visual field with trials in which items were presented in different visual fields. This analysis yielded a significant time interval (50–950 ms after stimulus onset) during which sensory-specific activity was processed during encoding (*T*_*sum*_ = 1337.00, *p*_*corr*_ = .020).

Second, we conducted pattern similarity analysis across the time window identified in the first analysis step to investigate the topographical distribution of sensory-specific activity. EEG data for each electrode was correlated across time (see Fig 2A). We subtracted the resulting neural pattern similarity of targets presented in the left visual field from the neural pattern similarity of targets presented in the right visual field. This procedure allowed us to “backproject” the EES differences for same versus different hemifields to the left and right hemisphere, allowing for the identification of the most sensitive electrodes in each hemisphere. According to this comparison, positive values marked electrodes primarily representing sensory representations of targets presented in the left visual field and negative values represented sensory processing in the right visual field. We therefore computed a lateralization score as the sum of the absolute *t*-sum values in the highest positive and negative cluster. The resulting lateralization score was tested against a permutation distribution shuffling item-pair similarity values of left and right visual field conditions 1,000 times. As expected, this analysis showed that electrode clusters in the right hemisphere showed higher similarity between items presented in the contralateral, left hemifield and vice versa, which was significantly more pronounced in the empirical data compared to the permutation distribution (*T*_*sum*_ = 24.59, *p*_*corr*_ = .013; see Fig 2A). These electrode clusters were selected to compute ERS values in the final step. Notably, this multivariate approach allowed us to track electrodes processing visual information from the contralateral hemifield, while univariate approaches comparing alpha/beta power between contra- and ipsilateral hemifields used in previous studies [37,38] did not show lateralized processing patterns during encoding in our study (see S9 Text and S8 Fig) and therefore were not suitable to measure reinstatement of sensory representations during memory retrieval.

#### Sensory feature reactivation analysis.

We calculated ERS using the approach described above in the time period from 150 ms to 1,350 ms after stimulus onset to ensure that all selected time windows only included time points measured in the time period of interest (0 ms to 1500 ms) – i.e., the time window 150 ms includes all time points from 0 ms to 300 ms and time window 1,350 consists of all time points from 1200 ms to 1500 ms. Notably, this approach results in a baseline period including time windows from both pre- and post-cue data points, which may result in significant clusters extending into baseline periods, e.g., the time window centered at time point 0s includes eight data points before cue onset and eight data points following cue onset. ERS values of objects presented in the same visual field during encoding and retrieval (same VF ERS) were contrasted to ERS of objects presented in different visual fields (different VF ERS). This was done using the selected respective contralateral electrodes, i.e., ERS for retrieval trials of objects presented in the left visual field to all encoding trials was computed over the selected right-hemispheric electrodes and vice versa for right visual field retrieval trials. These ERS patterns were averaged across trials to obtain mean measures of same VF ERS and different VF ERS. We first contrasted same VF ERS to different VF ERS for each retrieval condition and then contrasted this difference between involuntary and voluntary retrieval.

#### Item-specific reactivation analysis.

To analyze the reinstatement of item-specific activity during involuntary and voluntary retrieval, ERS was analyzed across all 64 electrodes in the 150 ms to 1,350 ms time window, assuming that item-specific information is characterized by a widespread pattern of EEG activity that is not confined to lateralized activity at contralateral sensor sites. In order to isolate item-specific activity, we compared ERS of same items (same item ERS) to ERS of different items presented in the same visual field (different VF ERS). We first compared same item ERS versus same VF ERS within each retrieval condition and then contrasted effects between conditions.

#### Reactivation compression.

We tested whether involuntary and voluntary reinstatement differed in their temporal dynamics. We quantified reactivation compression at the level of individual participants (Fig 3B). Within each participant, we contrasted same VF ERS to different VF ERS during involuntary retrieval, and same item ERS to same VF ERS during voluntary retrieval using two-sample *t*-tests across trials. Clusters were defined as adjacent encoding/retrieval time points with *t*-values above a subject-specific threshold (see below). We selected the cluster with the highest sum of *t*-values for each contrast and calculated participant-level reactivation compression for both clusters as the number of all time points per cluster divided by the number of retrieval time points (*RC = cluster size*/*retrieval time*). As a result, we obtained an involuntary and a voluntary reactivation compression score for each participant which we compared using paired *t*-tests. Subject-specific thresholds were defined as the mean of all positive *t*-values. We repeated the reactivation compression analysis with alternative thresholds to assess the robustness of effects. Indeed, we found the same results after increasing subject specific thresholds by adding 0.5 SDs, 1 SD or 1.5 SDs to the mean of all positive *t*-values (S3 Fig).

### Time-frequency analysis of induced theta power

#### Time-frequency analysis on electrode-level.

We estimated induced theta power after first subtracting the event-related potential calculated for each condition from the raw data [25]. Following this step, data were convolved with Morlet wavelets (width = 5), yielding a time-frequency representation from 2 to 30 Hz and −500–1,500 ms around stimulus onset. To analyze event-related power changes, post-stimulus data were baseline-corrected in relation to a −500 to −100 ms pre-stimulus period [25]. Consistent with previous research, theta was defined as the frequency band from 2 to 8 Hz [25,49].

Statistical analyses were conducted on the mean power of the a priori defined theta-band employing cluster-based permutation tests during the time window of 0–1,500 ms [57,58]. In a first step, a two-sided paired *t* test was calculated over all electrodes and time points between conditions. Spatially and temporally adjacent values exceeding the defined threshold of *p* < .05 created a cluster. The sum of *t*-values of each cluster represented the test statistic. Monte Carlo randomization was then used to estimate a distribution of the test statistic under the null hypothesis using 10,000 permutations. We used this test in order to assess differences between fully remembered hits and correctly rejected targets for the voluntary and the involuntary retrieval condition.

#### Source localization of theta power effects.

Source localization was accomplished using a linearly constrained minimum variance (LCMV) beamformer [42,96] on the whole time window of each trial as implemented in FieldTrip. Prior to LCMV beamforming a low-pass filter of 25 Hz was applied to the EEG. EEG sensor coordinates were estimated applying the JANUS toolbox [97] on a 3D head model of each subject calculated with Agisoft PhotoScan software (Version 1.3.2, Agisoft, LLC, St. Petersburg, Russia, www.agisoft.com) using pictures of the head from a 360° perspective. These electrodes were aligned with a representative structural T1-weighted MR image and then used to construct individual head models. The boundary element method (BEM) was applied to estimate volume conduction during forward modelling based on a standard BEM model distributed by the fieldtrip toolbox. Source positions were arranged on a 10 mm grid covering the whole brain comparted by the BEM. Individual head models and the standard BEM model were normalized to MNI space and finally entered into the LCMV beamformer.

The resulting EEG on source level was transformed into a time-frequency representation as done with the EEG on the scalp level but at the virtual sensors in source space. We conducted source analyses for the two comparisons of the time frequency data described on sensor level. For each source analysis, the mean power in the theta band was averaged over the significant time window revealed by the corresponding time-frequency analysis on sensor level (involuntary retrieval: 0−1,500 ms; voluntary retrieval: 450−1,350 ms). We performed non-parametric cluster statistics to analyze the spatial distribution of the source data. A cluster consisted of adjacent sources exceeding the cluster threshold of *p* < .005. As in the previously described cluster statistics, the test statistic results from the sum of the cluster *t*-values and is compared to an estimated random distribution across 10,000 permutations using a two-sided one-sample *t* test. Notably, source analyses were conducted in order to follow-up the results already evident at scalp level and to describe peak sources of these scalp effects in more detail [84,98]. Therefore, we consider trend clusters as relevant peak sources underlying theta-power effects on scalp level.

### Effect sizes for RSA and theta-band results

We reported effect sizes for all significant results in cluster permutation statistics as ranges between their lower and upper bounds following fieldtrip guidelines [99]. The lower bound of the effect size was calculated by defining a rectangle around a significant cluster which was used to compute the mean difference between conditions in each participant. This was used to calculate Cohen’s *d*. The upper bound of the effect size was calculated as the maximum Cohen’s *d* value at an individual data point within the original cluster.

## Supporting information

S1 Fig Behavioral results in EEG sample. Response rates, response bias and response times during visual discrimination (involuntary retrieval cover task) and voluntary retrieval task. Error bars reflect standard errors of the mean.* *p* < .05; Inv, involuntary; Vol, voluntary.
(TIF)

S2 Fig Item-specific reactivation in posterior regions of interest. **A.** Left posterior electrodes. **B.** Right posterior electrodes. Left: Involuntary retrieval. Middle: Voluntary retrieval. Right: Interaction effects. Note: This figure illustrates results that are not significant following multiple comparison corrections for the six regions of interest.
(TIF)

S3 Fig Results of three-dimensional cluster analysis. Left: Encoding time x retrieval time plot of RSA results averaged across all electrodes from a cluster showing item-specific reactivation during voluntary retrieval at trend level (*p*_*corr*_ = .089). Black lines indicate baseline period. The baseline period includes time windows from both pre- and post-cue data points which may account for the apparent pre-cue effects. For example, the time window centered at time point 0s includes eight data points before cue onset and eight data points following cue onset. Therefore, these data points do not represent noise or pre-cue results. Right: Topographical plot of RSA results. Significant electrodes are marked as larger black dots. Note, this figure illustrates a cluster showing a non-significant trend effect (*p*_*corr*_ = .089).
(TIF)

S4 Fig Reactivation compression analysis within participants using different cluster thresholds. Involuntary reactivation compression was calculated for the contrast same VF versus different VF ERS, and voluntary reactivation was calculated for the contrast of same item versus same VF ERS. Cluster thresholds were defined for each participant individually as **A.** Mean (M); **B.** Mean + 0.5*standard deviation of the mean (SD); **C.** Mean + standard deviation of the mean; **D**. Mean + 1.5*standard deviations of the mean of all positive reactivation values. Raincloud plots represent group distributions, individual data points and boxplot including median. * *p* < .05.
(TIF)

S5 Fig Variance in peak latencies of memory reactivation during involuntary and voluntary memory retrieval. Raincloud plots represent group distributions, individual data points and boxplot including median. N.s. – not significant (*p* > .05).
(TIF)

S6 Fig Power differences in slow-theta (2–4 Hz) and fast-theta (4–8 Hz). **A.** Involuntary retrieval slow-theta power effects. Left, top: time course of slow-theta power in significant electrodes. *T*-sum values above zero indicate significant time points. Left, bottom: time-frequency plot of slow-theta activity as mean across all significant electrodes. Time-frequency plots are only displayed for illustrative purposes and were not used for inference statistics. Right: topographical plot of significant electrode clusters. Significant electrodes are highlighted as bold dots. **B.** Voluntary retrieval slow-theta power effects. **C.** Involuntary retrieval fast-theta power effects. **D.** Voluntary retrieval fast-theta power effects.
(TIF)

S7 Fig Left, Power spectra for theta, alpha and beta frequency bands. Middle, theta abundance results comparing full-hits versus correct rejections during involuntary retrieval and aperiodic slope results comparing full-hits versus correct rejections during involuntary retrieval. Negative values indicate steeper slopes. Right, theta abundance and aperiodic slope results in the voluntary retrieval phase. Larger black dots indicate significant electrodes.
(TIF)

S8 Fig Time-frequency analysis comparing encoding of items presented in the left or right visual hemifield. Mean *t*-statistics during the time window 0.2 s–0.7 s over alpha/beta frequencies (8–20 Hz) which has previously been shown to comprise lateralized power decreases [38].
(TIF)

S1 Text Behavioral results in EEG sample. (PDF)

S2 Text Full-hits to hits ratio. (PDF)

S3 Text Item-specific reactivation analysis in anatomically defined brain regions. (PDF)

S4 Text Item-specific reactivation analysis in three-dimensional spatiotemporal cluster approach. (PDF)

S5 Text Within-subject analysis of memory compression using different thresholds. (PDF)

S6 Text Analysis of variability of memory reactivation timings. (PDF)

S7 Text Theta-power in slow- and fast-theta frequency bands. (PDF)

S8 Text Effects in theta frequency band controlled for aperiodic slope. (PDF)

S9 Text Analyzing alpha/beta power decreases in contralateral electrodes during encoding. (PDF)

S1 Table Supplementary table. (DOCX)

## Abbreviations

ACC: anterior cingulate cortex
BEM: boundary element method
EEG: electroencephalography
EES: encoding-encoding similarity
ERS: encoding-retrieval similarity
FA: false alarms
ICA: independent component analysis
LCMV: linearly constrained minimum variance
PTSD: posttraumatic stress disorder
