# Supplementary figures and images for: Involuntary and voluntary memory retrieval relies on distinct neural representations and oscillatory processes
Source: PLoS Biol. 2025 Aug 19;23(8):e3003258. doi: 10.1371/journal.pbio.3003258 (PMC12364361; doi:10.1371/journal.pbio.3003258)

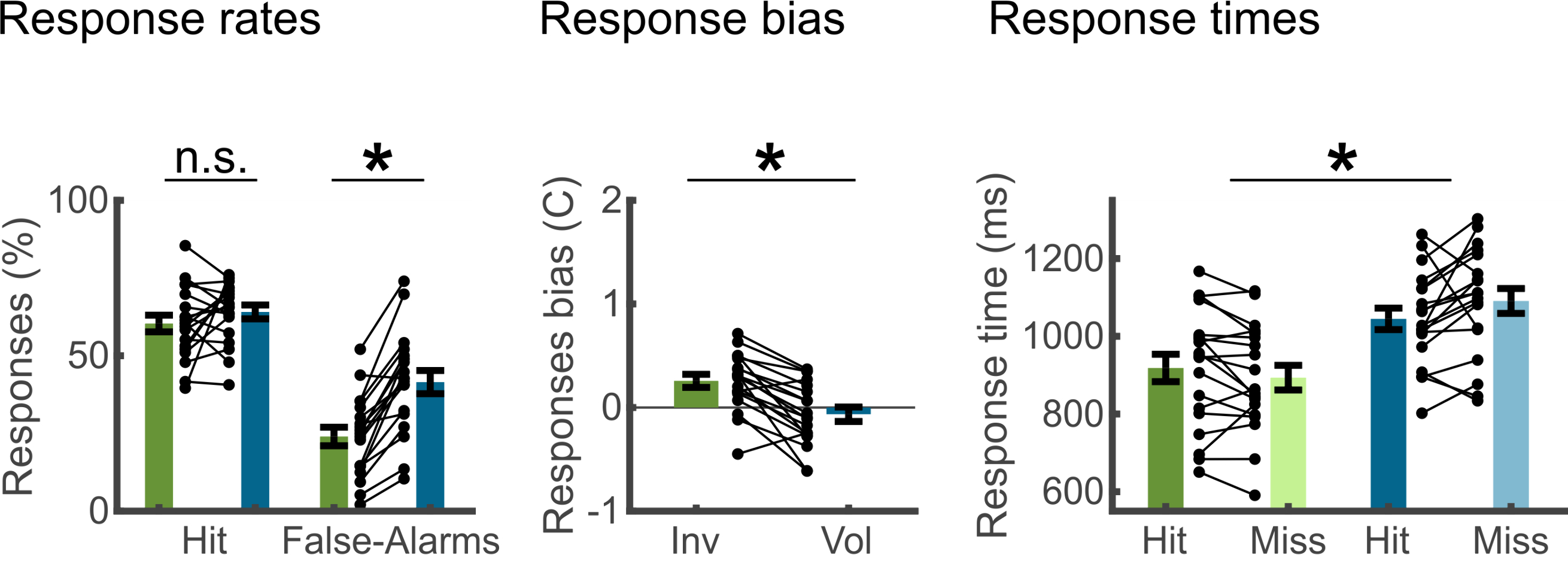

Supplement: S1 Fig — Response rates, response bias and response times during visual discrimination (involuntary retrieval cover task) and voluntary retrieval task. Error bars reflect standard errors of the mean.* p < .05; Inv, involuntary; Vol, voluntary. (TIF) [file pbio.3003258.s001.tif]

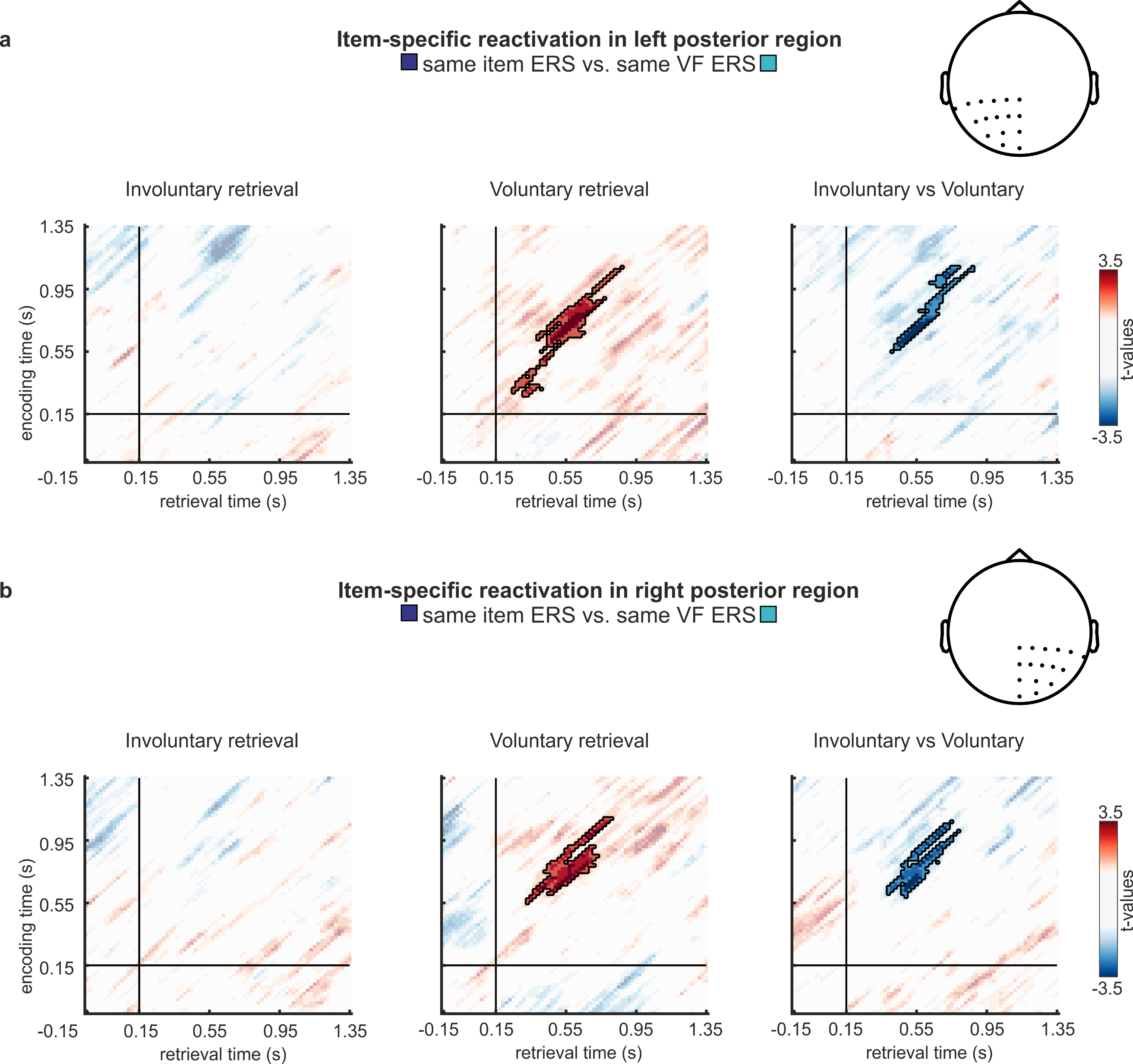

Supplement: S2 Fig — A. Left posterior electrodes. B. Right posterior electrodes. Left: Involuntary retrieval. Middle: Voluntary retrieval. Right: Interaction effects. Note: This figure illustrates results that are not significant following multiple comparison corrections for the six regions of interest. (TIF) [file pbio.3003258.s002.tif]

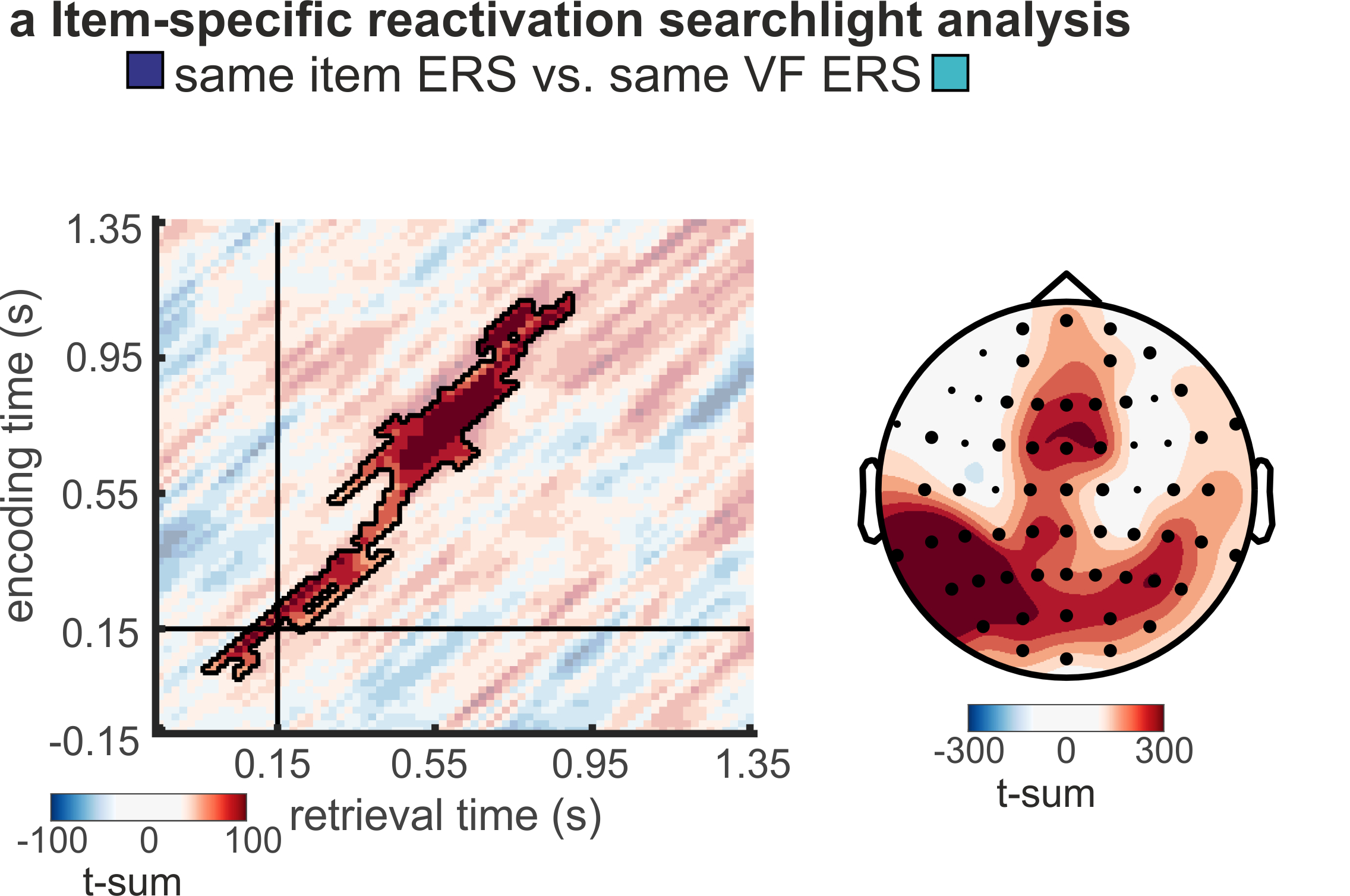

Supplement: S3 Fig — Left: Encoding time x retrieval time plot of RSA results averaged across all electrodes from a cluster showing item-specific reactivation during voluntary retrieval at trend level (pcorr = .089). Black lines indicate baseline period. The baseline period includes time windows from both pre- and post-cue data points which may account for the apparent pre-cue effects. For example, the time window centered at time point 0s includes eight data points before cue onset and eight data points following cue onset. Therefore, these data points do not represent noise or pre-cue results. Right: Topographical plot of RSA results. Significant electrodes are marked as larger black dots. Note, this figure illustrates a cluster showing a non-significant trend effect (pcorr = .089). (TIF) [file pbio.3003258.s003.tif]

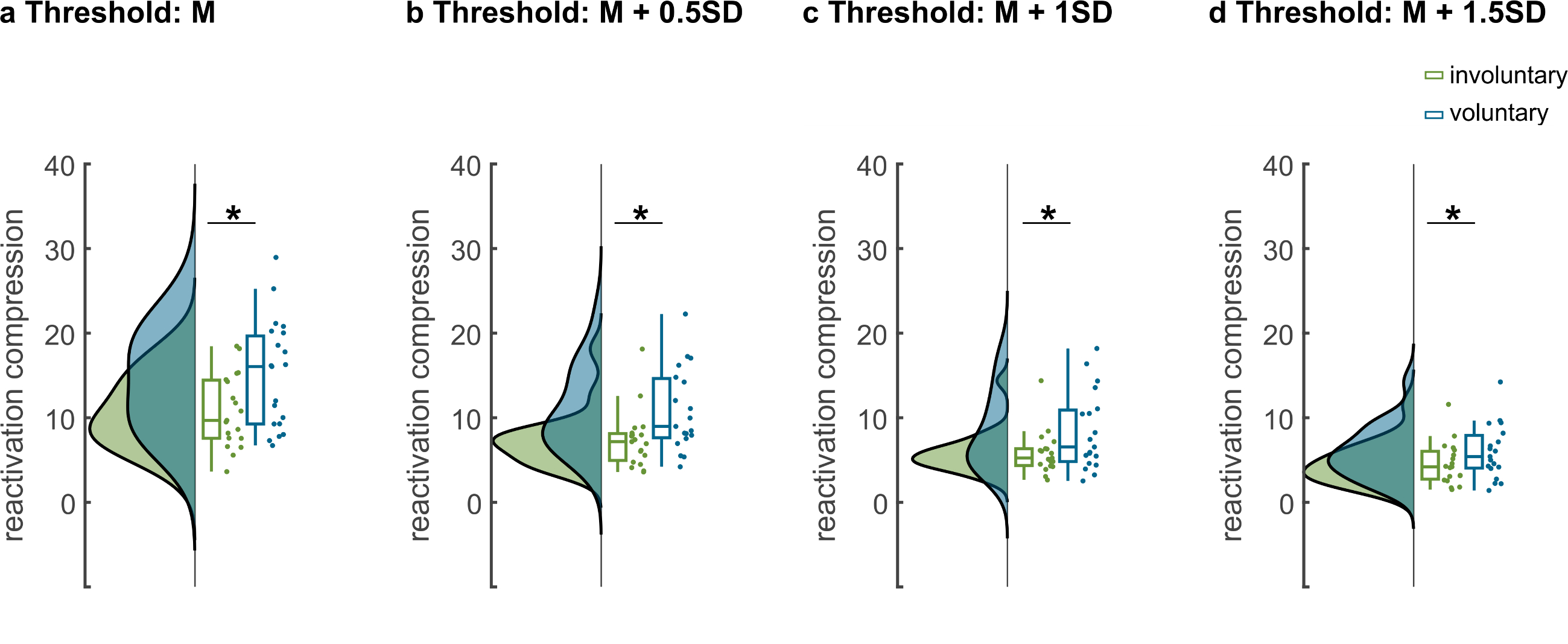

Supplement: S4 Fig — Involuntary reactivation compression was calculated for the contrast same VF versus different VF ERS, and voluntary reactivation was calculated for the contrast of same item versus same VF ERS. Cluster thresholds were defined for each participant individually as A. Mean (M); B. Mean + 0.5*standard deviation of the mean (SD); C. Mean + standard deviation of the mean; D. Mean + 1.5*standard deviations of the mean of all positive reactivation values. Raincloud plots represent group distributions, individual data points and boxplot including median. * p < .05. (TIF) [file pbio.3003258.s004.tif]

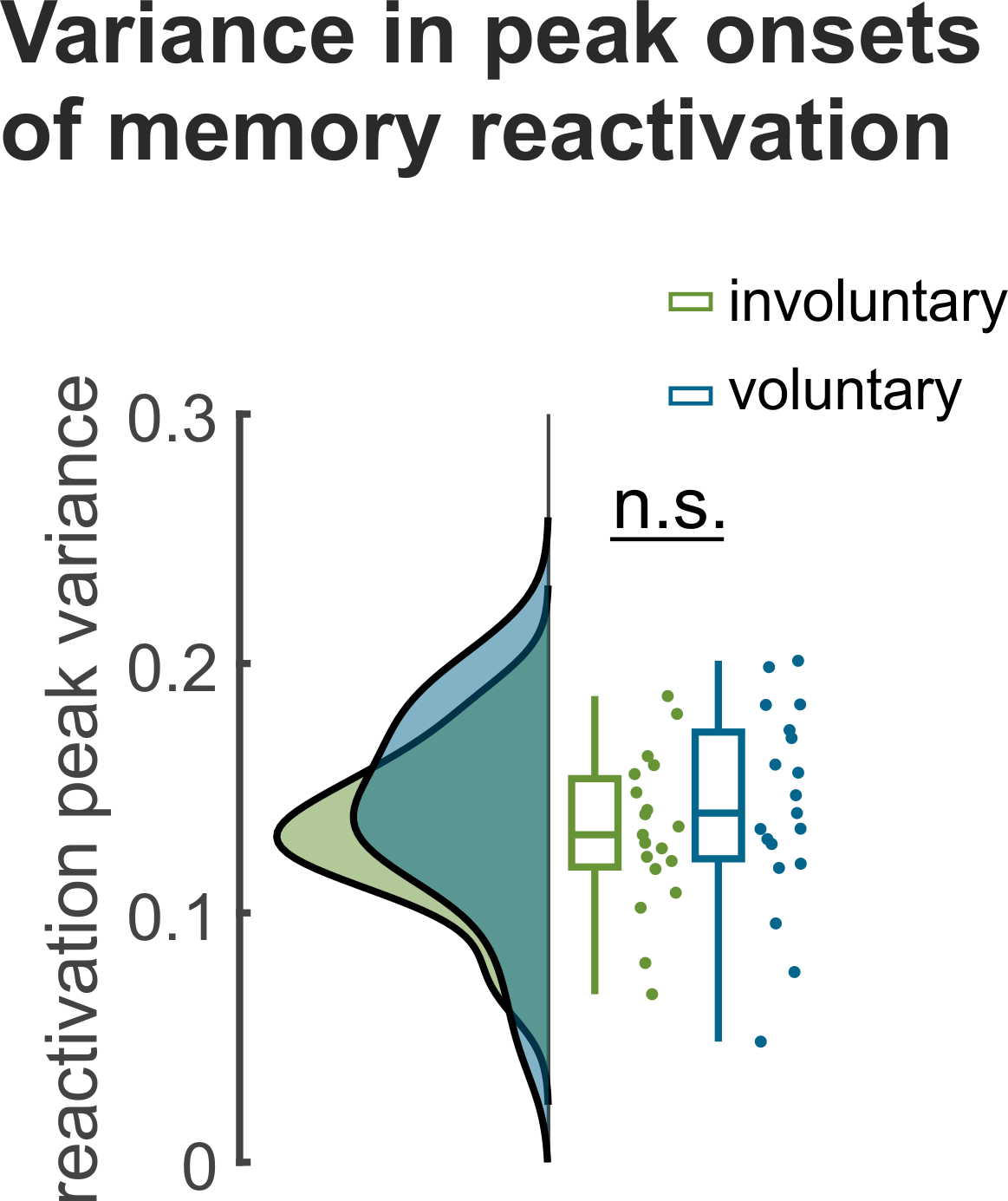

Supplement: S5 Fig — Raincloud plots represent group distributions, individual data points and boxplot including median. N.s. – not significant (p > .05). (TIF) [file pbio.3003258.s005.tif]

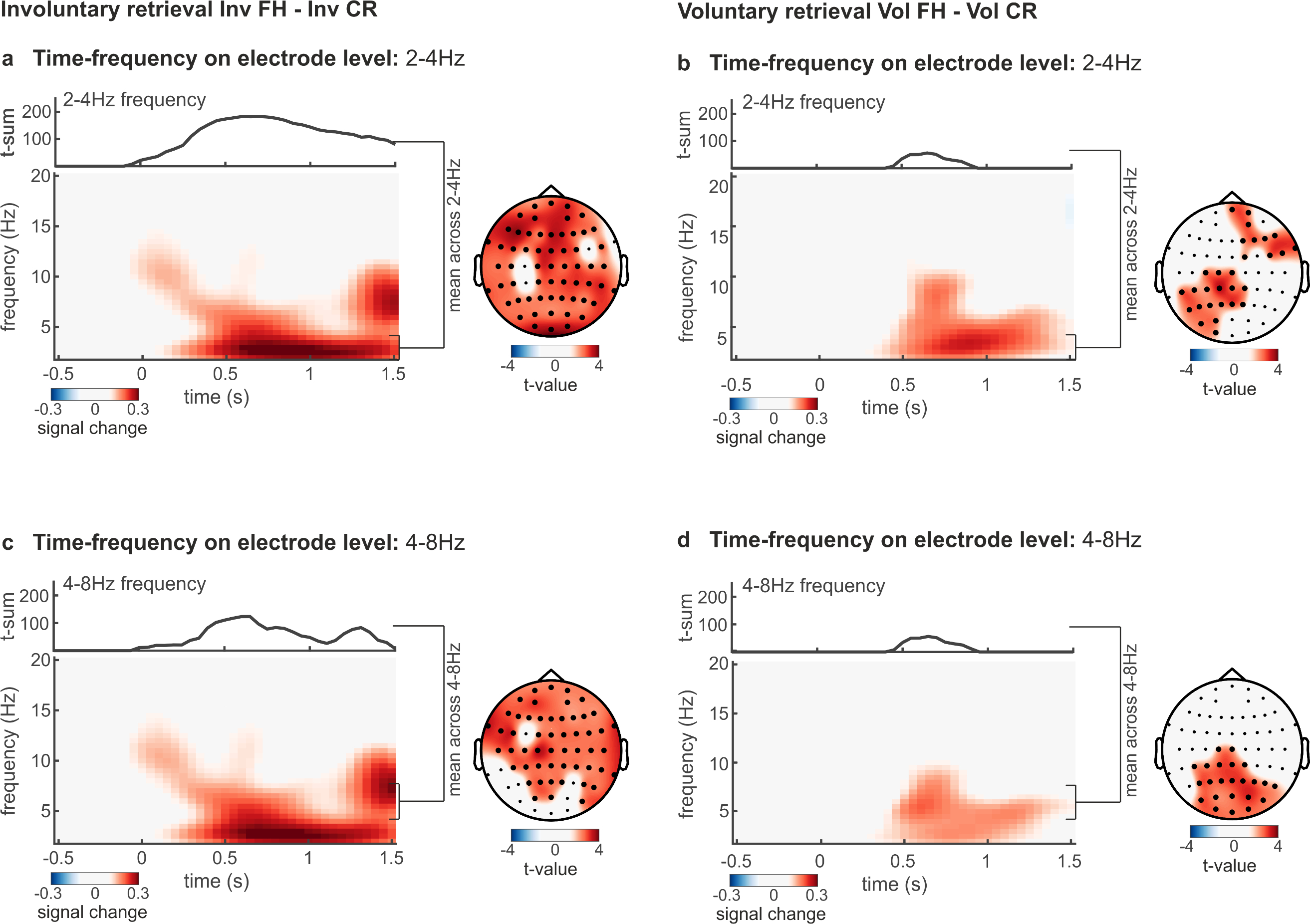

Supplement: S6 Fig — A. Involuntary retrieval slow-theta power effects. Left, top: time course of slow-theta power in significant electrodes. T-sum values above zero indicate significant time points. Left, bottom: time-frequency plot of slow-theta activity as mean across all significant electrodes. Time-frequency plots are only displayed for illustrative purposes and were not used for inference statistics. Right: topographical plot of significant electrode clusters. Significant electrodes are highlighted as bold dots. B. Voluntary retrieval slow-theta power effects. C. Involuntary retrieval fast-theta power effects. D. Voluntary retrieval fast-theta power effects. (TIF) [file pbio.3003258.s006.tif]

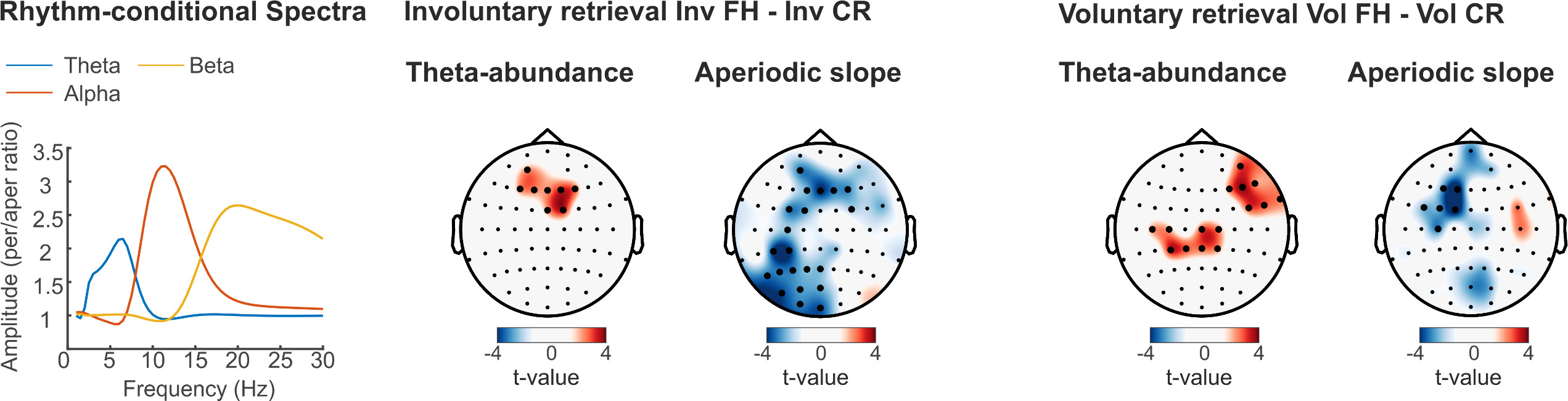

Supplement: S7 Fig — Middle, theta abundance results comparing full-hits versus correct rejections during involuntary retrieval and aperiodic slope results comparing full-hits versus correct rejections during involuntary retrieval. Negative values indicate steeper slopes. Right, theta abundance and aperiodic slope results in the voluntary retrieval phase. Larger black dots indicate significant electrodes. (TIF) [file pbio.3003258.s007.tif]

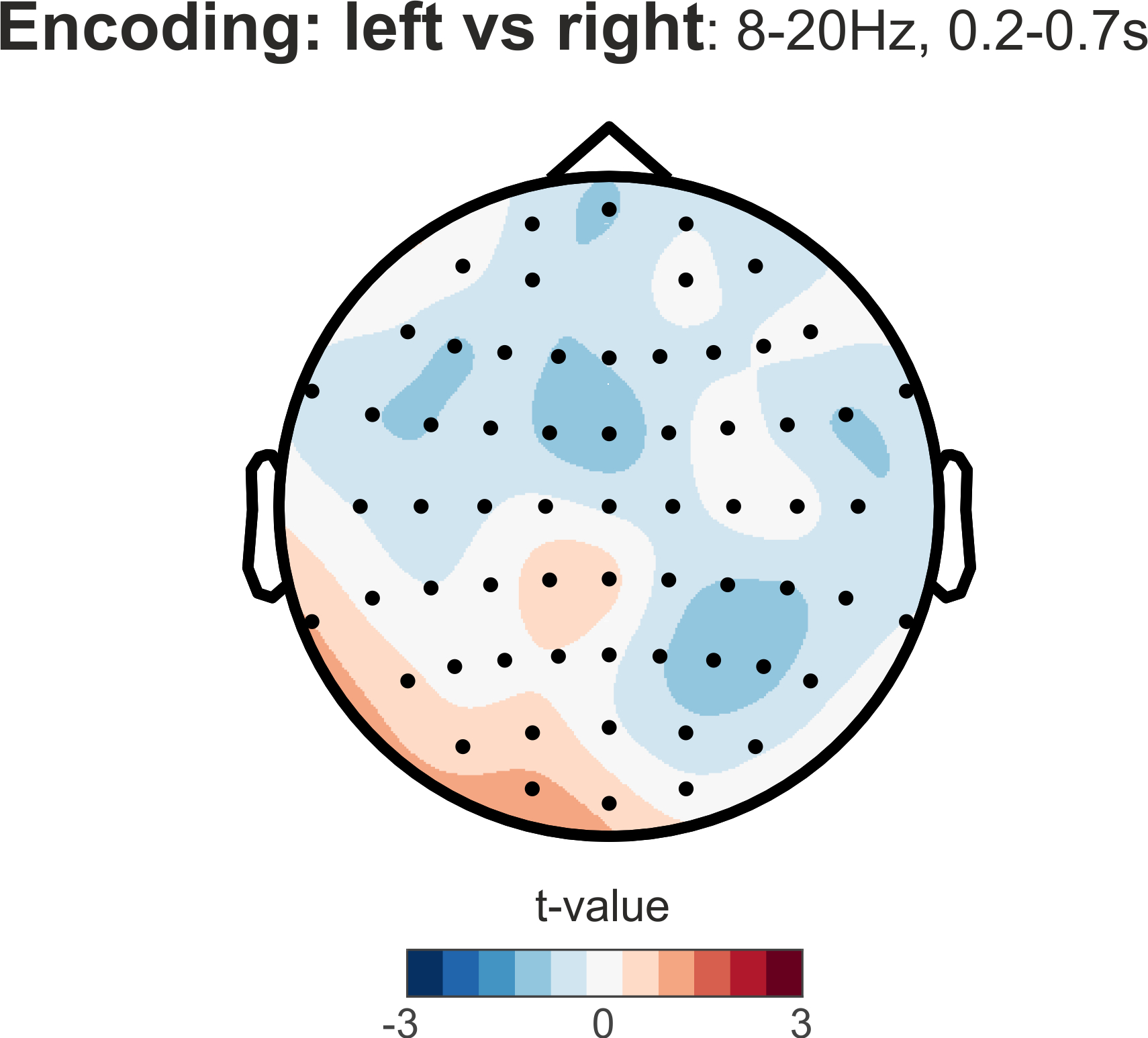

Supplement: S8 Fig — Mean t-statistics during the time window 0.2 s–0.7 s over alpha/beta frequencies (8–20 Hz) which has previously been shown to comprise lateralized power decreases [38]. (TIF) [file pbio.3003258.s008.tif]
