# Supplementary material for: Involuntary and voluntary memory retrieval relies on distinct neural representations and oscillatory processes
Source: PLoS Biol. 2025 Aug 19;23(8):e3003258. doi: 10.1371/journal.pbio.3003258 (PMC12364361; doi:10.1371/journal.pbio.3003258)
Supplement: S1 Text — (PDF) [file pbio.3003258.s009.pdf]

S1 Text. Behavioral results in EEG sample

We calculated all EEG analyses in a subsample of 19 participants (age: $M = 24$, $SD = 4.55$; 10 females). Behavioral findings in this EEG subsample were in line with behavioral results in the whole participant sample ($N = 31$). Memory performance was better during involuntary than voluntary retrieval (involuntary retrieval: $M = 1.07$, $SD = 0.65$; voluntary retrieval: $M = 0.61$, $SD = 0.45$; $t_{18} = 6.16$, $p < .001$, $d = 1.41$; fig. S1). This effect was driven by lower false-alarm rates during involuntary retrieval ($t_{18} = 7.77$, $p < .001$, $d = 1.78$), while we found no difference in hit-rates ($t_{18} = -1.34$, $p = .198$, $d = 0.31$). Memory responses were more conservative during involuntary than voluntary retrieval (involuntary retrieval: $M = 0.26$, $SD = 0.28$; voluntary retrieval: $M = -0.07$, $SD = 0.30$; $t_{18} = 5.46$, $p < .001$, $d = 1.25$).

We furthermore tested whether involuntary retrieval interfered with the visual discrimination task using a 2 (retrieval condition) x 2 (memory: hits vs miss) ANOVA. Response times were slower during involuntary than voluntary retrieval ($F_{1,18} = 26.70$, $p < .001$, $\eta_p^2 = 0.60$), but no difference in response times between hits and misses was found ($F_{1,18} = 0.76$, $p = .394$, $\eta_p^2 = 0.04$). Crucially, we found a significant condition x memory interaction ($F_{1,18} = 5.34$, $p = .033$, $\eta_p^2 = 0.23$) indicating that differences in response times between hits and misses were modulated by involuntary and voluntary retrieval tasks. Yet, post-hoc t-tests did not demonstrate a significant difference between response times during involuntary hits and misses ($t_{18} = 1.70$, $p = .106$, $d = 0.39$) or between voluntary hits and misses ($t_{18} = -1.96$, $p = .065$, $d = 0.45$).

We additionally conducted post-hoc analyses of reaction times during source and forced-choice item memory tasks. One may argue that differences in response times during these tasks may reflect differences in accessibility of memory content during the preceding cue presentation period. For example, faster response times for correct source memory trials during involuntary compared to voluntary memories may suggest that involuntary memories retrieved source memory information already during cue presentation, while voluntary memories required additional retrieval effort to access source memory content during the respective memory task. However, we acknowledge that our paradigm is not particularly well-suited to test this hypothesis. First, memory retrieval processes were likely to be completed before responding to both memory tasks as cue presentation onset started two seconds before both memory tasks. Second, participants were not instructed to answer memory tasks as fast as possible and had no time pressure to answer the task as they were given five seconds to answer them. Third, trial number of misses in the forced-choice item memory task during involuntary memories was very low with six participants having no misses in this task and only three participants with more than ten trials.

We observed faster response times for hits compared to misses in source ($t_{17} = -3.70$, $p = .002$, $d = 0.87$) and forced-choice item recognition tasks ($t_{17} = -2.94$, $p = .009$, $d = 0.69$) during voluntary retrieval. During involuntary retrieval, we also found faster reaction times for hits compared to misses in the source memory task ($t_{17} = -2.41$, $p = .028$, $d = 0.57$), while this effect was not significant in the forced-choice item recognition task ($t_{17} = -0.98$, $p = .341$, $d = 0.23$). Involuntary and voluntary retrieval did not differ in response times (hits vs misses) during source ($t_{17} = 1.53$, $p = .146$, $d = 0.36$) or forced-choice item recognition tasks ($t_{17} = 0.51$, $p = .615$, $d = 0.12$).
