# Supplementary material for: Involuntary and voluntary memory retrieval relies on distinct neural representations and oscillatory processes
Source: PLoS Biol. 2025 Aug 19;23(8):e3003258. doi: 10.1371/journal.pbio.3003258 (PMC12364361; doi:10.1371/journal.pbio.3003258)
Supplement: S2 Text — (PDF) [file pbio.3003258.s010.pdf]

S2 Text. Full-hits to hits ratio

The higher amount of false-alarms during voluntary compared to involuntary retrieval may suggest that voluntary memories were more accessible than involuntary memories which may be surprising given that involuntary memories are characterized by their spontaneous retrieval following generalized memory cues. Yet, more false-alarms can occur due to two reasons: higher accessibility, or a more liberal response criterion. Our findings suggest that voluntary retrieval was associated with a more liberal response criterion compared to involuntary retrieval (see main text). To underline this assumption, we tested the ratio of full-hits to hits in both conditions as memory responses under uncertainty should be related to less recollection and thus less accurate and detailed target memories. Indeed, we observed that the full-hits to hits ratio was lower during voluntary than involuntary memory retrieval (M_{inv} = 0.50, SD_{inv} = 0.15; M_{vol} = 0.40, SD_{vol} = 0.16; t_{30} = 4.61; p < .001), suggesting that during voluntary retrieval participants were more likely to indicate remembering a cue when it was familiar even without remembering associated target information. This more liberal response criterion during voluntary retrieval was adaptive as it was an optimal strategy for the recognition test to indicate a memory every time they were a little bit more certain that an item was old than new. In contrast, the involuntary retrieval phase was no memory test and therefore participants did not intend to most accurately respond to the involuntary memory question but only reported involuntary memories when they undoubtedly intruded into their mind. Thus, our findings of more false alarms during voluntary retrieval do not contradict generalized accessibility of involuntary memories as they may be explained by a more liberal response criterion during voluntary retrieval.
