# Supplementary material for: Involuntary and voluntary memory retrieval relies on distinct neural representations and oscillatory processes
Source: PLoS Biol. 2025 Aug 19;23(8):e3003258. doi: 10.1371/journal.pbio.3003258 (PMC12364361; doi:10.1371/journal.pbio.3003258)
Supplement: S3 Text — (PDF) [file pbio.3003258.s011.pdf]

S3 Text. Item-specific reactivation analysis in anatomically defined brain regions

To identify subregions showing item-specific reactivation, we conducted item-specific reactivation analyses in six electrode clusters (frontal left, frontal right, middle left, middle right, posterior left, posterior right). Electrode clusters were defined based on previous studies using similar RSA approaches (1). For each electrode cluster, we calculated item-specific reactivation by comparing within-item representational similarity vs between-item similarity for voluntary and involuntary retrieval and finally contrasted effects in both conditions to test for interaction effects.

We found no significant item-specific reactivation during involuntary retrieval in any of the predefined electrode clusters (all p_{corrs} > .108). During voluntary retrieval, we observed item-specific reactivation in left posterior electrodes with a cluster spanning from 270ms to 1090ms during encoding and from 250ms to 870ms during retrieval (fig. S2a). Moreover, we observed item-specific reactivation in right posterior electrodes with a cluster from 550ms to 1090ms during encoding and from 330ms to 810ms during retrieval (fig. S2b). However, effects in both regions did not survive multiple comparison correction in the six anatomical ROIs (left posterior region: p_{corr} = .108; right posterior region: p_{corr} = .240). In both regions, we observed trends that item-specific reactivation was higher during voluntary than involuntary retrieval (right posterior regions: t_{sum} = -356.92, p_{corr} = .051, d_{lower} = 0.62, d_{upper} = 0.91; left posterior regions: t_{sum} = -285.75, p_{corr} = .099, d_{lower} = 0.50, d_{upper} = 1.09). Both effects did not survive multiple comparison correction for the six ROIs (left posterior region: p_{corr} = .594; right posterior region: p_{corr} = .301). We did not find significant effects in any other region (all p_{corrs} > .544).
