# Supplementary material for: Involuntary and voluntary memory retrieval relies on distinct neural representations and oscillatory processes
Source: PLoS Biol. 2025 Aug 19;23(8):e3003258. doi: 10.1371/journal.pbio.3003258 (PMC12364361; doi:10.1371/journal.pbio.3003258)
Supplement: S4 Text — (PDF) [file pbio.3003258.s012.pdf]

S4 Text. Item-specific reactivation analysis in three-dimensional spatiotemporal cluster approach

As a complementary, data-driven procedure to assess item-specific reactivation, we developed a three-dimensional searchlight approach. The z-transformed EEG data were segmented into overlapping time windows of 300ms with increments of 20ms as in the previous RSA analyses. Crucially, for each electrode, neighboring channels were defined using the triangulation method in fieldtrip and the resulting matrix of 16 time points x n channels was concatenated for each electrode. For each electrode, the resulting vectors during encoding were then correlated with all vectors during the retrieval period using Spearman's correlations and were then Fisher-z-transformed. This results in three-dimensional ERS matrices for all combinations of encoding time bins and retrieval time bins across electrodes. For statistical testing, two-sided cluster-based permutation statistics implemented in fieldtrip were used to correct for multiple comparisons.

Using this approach, we observed for voluntary retrieval a trend for reactivation of item-specific representations in an encoding time period from 150ms to 890ms and during retrieval from 150ms to 1130ms (t_{sum} = 8354.67; p_{corr} = .089; fig. S3). This effect was observed in a widespread cluster including 54/64 electrodes (84.38% of electrodes) with a peak in posterior temporoparietal electrodes. We did not observe any item-specific reactivation during involuntary retrieval (p_{corr} > .651) or any interactions between voluntary and involuntary reactivation (p_{corr} > .542).
