# Supplementary material for: Involuntary and voluntary memory retrieval relies on distinct neural representations and oscillatory processes
Source: PLoS Biol. 2025 Aug 19;23(8):e3003258. doi: 10.1371/journal.pbio.3003258 (PMC12364361; doi:10.1371/journal.pbio.3003258)
Supplement: S5 Text — (PDF) [file pbio.3003258.s013.pdf]

**S5 Text. Within-subject analysis of memory compression using different thresholds**

We found higher memory compression during voluntary than involuntary retrieval using a within-subject analysis (see fig. S4). This analysis was based on identifying the largest reinstatement clusters during both retrieval modes using an individual threshold within each subject to detect reinstatement time points. The individual threshold of each participant was calculated as the mean of all positive encoding-retrieval similarity values of each contrast. To control that our findings were not the result of the selection of this particular threshold, we repeated the same analysis with other thresholds by adding 0.5, 1 or 1.5 standard deviations to the mean value of all positive encoding-retrieval similarity values (T-value thresholds involuntary retrieval: $M$: 0.82 ± 0.21, $M + 0.5SD$: 1.13 ± 0.27, $M + 1SD$: 1.44 ± 0.33, $M + 1.5SD$: 1.75 ± 0.40; t-value thresholds voluntary retrieval: $M$: 0.84 ± 0.21, $M + 0.5SD$: 1.15 ± 0.28, $M + 1SD$: 1.46 ± 0.36, $M + 1.5SD$: 1.77 ± 0.44). We found that memory compression was higher for voluntary than involuntary memory retrieval when repeating the analysis with each threshold (threshold $M + 0.5SD$: $t_{18} = 4.44$, $p < .001$, 95% CI = [1.84, 5.10], $d = 1.03$; threshold $M + 1SD$: $t_{18} = 2.92$, $p = .009$, 95% CI = [0.76, 4.39], $d = 0.68$; threshold $M + 1.5SD$: $t_{18} = 2.78$, $p = .012$, 95% CI = [0.35, 2.53], $d = 0.64$; fig. S4).
