# Supplementary material for: Involuntary and voluntary memory retrieval relies on distinct neural representations and oscillatory processes
Source: PLoS Biol. 2025 Aug 19;23(8):e3003258. doi: 10.1371/journal.pbio.3003258 (PMC12364361; doi:10.1371/journal.pbio.3003258)
Supplement: S6 Text — (PDF) [file pbio.3003258.s014.pdf]

S6 Text. Analysis of variability of memory reactivation timings

Reactivation compression may be confounded by variations in the timing of memory reactivation. Specifically, one may argue that onsets of involuntary memory reactivation may be more variable due to their uncontrolled nature which may result in artificial detection of more extended temporal clusters. We therefore analyzed the variability of peak latencies of memory reactivation. If involuntary memory reactivation occurs at more variable time points, this should be reflected in a higher variability of the latencies of memory reactivation peaks during involuntary than voluntary retrieval.

To test this hypothesis, we extracted the latencies of memory reactivation peaks during involuntary and voluntary memories. For each involuntary memory trial, we identified the time point of maximum low-level sensory reactivation, i.e., the difference between same VF ERS and different VF ERS. Similarly, for each voluntary memory trial, we selected the time point of maximum item-specific reactivation, i.e. the difference between same item ERS and same VF ERS. We then calculated the variance of peak reactivation timing during involuntary and voluntary memory retrieval in each participant and compared variances between both conditions using a dependent t-test across participants. We found no difference in variances of peak latencies between involuntary and voluntary retrieval (t_{19} = -1.18, p = .254, fig. S5). In fact, variability of peak onsets was numerically lower during involuntary than voluntary memory retrieval.

We furthermore tested whether we could find evidence for differences in peak timing variability within participants. Within each participant, we applied a Levene-Test to compare variances between involuntary and voluntary peak latencies of memory reactivation. Out of 19 participants, only one participant showed a significant difference in variance ($F_{1,66}$ = 4.45, p = .039; all other ps > .137). In this participant, variability of peak latencies was even lower during involuntary than voluntary memories (Var_{inv} = 0.126 s^2, Var_{vol} = 0.199 s^2). Overall, peak latency analyses did not show evidence for higher variability of memory reactivation timings during involuntary compared to voluntary memories and therefore may not confound memory compression differences.
