# Supplementary material for: Involuntary and voluntary memory retrieval relies on distinct neural representations and oscillatory processes
Source: PLoS Biol. 2025 Aug 19;23(8):e3003258. doi: 10.1371/journal.pbio.3003258 (PMC12364361; doi:10.1371/journal.pbio.3003258)
Supplement: S7 Text — (PDF) [file pbio.3003258.s015.pdf]

S7 Text. Theta-power in slow- and fast-theta frequency bands

We defined theta oscillations in a broad frequency spectrum ranging from 2 to 8 Hz in line with previous studies suggesting that episodic memory retrieval is related to “slow theta” in the range of 2-4 Hz (2–6) and “fast-theta” in the range of 4-8 Hz (7). Notably, this aligns with the presented double-peak in low and high theta-frequencies in the spectral density plot of this experiment (see fig. S7). To more precisely understand whether our effects in theta-power can be related to slow-theta, fast-theta, or both, we conducted two complementary analyses defining theta-power in the range of 2-4 Hz and 4-8Hz.

In the slow-theta range, involuntarily remembered full-hits compared to correct rejections induced early theta-power increases starting at time point 0ms until 1500ms after cue onset with a widespread topography with peaks in midfrontal electrodes comparable to the results in the main manuscript (t_{sum} = 3844.44, p_{corr} = .004, d_{lower} = 1.00, d_{upper} = 1.12; see fig. S6a). Voluntarily retrieved full-hits were associated with increased theta-power ranging from 450ms to 1450ms after cue onset and including right frontal electrodes and centroparietal electrodes (t_{sum} = 732.49, p_{corr} = .026, d_{lower} = 0.85, d_{upper} = 0.99; fig. S6b), again largely overlapping with the main manuscript results.

In the fast-theta range (4-8Hz), during involuntary retrieval, full-hits compared to correct rejections induced early theta-power increases starting at time point 0 until 1.5s after cue onset with a midfrontal topography (t_{sum} = 1813.43, p_{corr} = .006, d_{lower} = 0.98, d_{upper} = 1.07, fig. S6c). During voluntary retrieval, full-hits compared to correct rejections were associated with higher theta-power in a cluster ranging from 450ms to 900ms and distributed across posterior electrodes (t_{sum} = 350.64, p_{corr} = .04, d_{lower} = 0.76, d_{upper} = 0.86; fig. S6d). These results largely align with the results in the slow-theta range, except that theta-power increases in lateral frontal electrodes during voluntary retrieval can only be observed in the slow-theta band.

Overall, although splitting theta oscillations into two smaller frequency bands may reduce the power of our analyses, we find largely overlapping clusters as in the analyses defining theta-bands from 2-8 Hz.
