# Supplementary material for: Involuntary and voluntary memory retrieval relies on distinct neural representations and oscillatory processes
Source: PLoS Biol. 2025 Aug 19;23(8):e3003258. doi: 10.1371/journal.pbio.3003258 (PMC12364361; doi:10.1371/journal.pbio.3003258)
Supplement: S8 Text — (PDF) [file pbio.3003258.s016.pdf]

S8 Text. Effects in theta frequency band controlled for aperiodic slope

Differences in oscillatory power can be confounded by differences in the aperiodic slope between conditions. We therefore used the eBOSC toolbox to dissociate oscillatory activity from aperiodic slope-related activity (8). This method defines rhythms as time points exceeding aperiodic activity with a duration of at least three cycles of a given frequency. Aperiodic activity was calculated by fitting a linear robust regression in log(frequency)-log(power) coordinates. To reduce confounding effects of alpha peak frequencies on arhythmic background estimates, individual peak frequencies were removed within the 8-15Hz range. The resulting arhythmic background estimate was used to test for oscillation periods as time periods exceeding the 95th percentile of a X^2 (2)-distribution of power values centered on the linearly fitted estimate of background power at the respective frequency. This approach was conducted for each channel in each trial and participant to detect trial-specific rhythmic time periods.

As a first sanity check, we used this approach to test whether our experiment across all trials induced reliable theta oscillations. For this purpose, we estimated averaged power estimates during all time periods detecting oscillations in a frequency range of 2-8Hz and divided these power values by averaged power estimates in this frequency range during all time periods that did not show oscillations in this frequency range. This approach reveals whether theta oscillations reliably induced higher power than aperiodic time periods. Using the same approach, we also checked for oscillations in the alpha (9-15Hz) and beta range (16-30Hz). In line with our assumption that the experimental task induced theta oscillations and previous findings that theta oscillations in humans occur both in a lower delta/theta and a higher theta band (7), we observed a double-peak scenario with a lower peak at 3 Hz and a larger peak at 6.5 Hz (fig. S7).

As a next step, we aimed to test whether differences in theta oscillations between full-hits and correct rejections in the voluntary and involuntary retrieval phase can be attributed to the rhythm duration (abundance) and/or the power of detected oscillations in the theta-frequency range. This approach thus allows to decompose the observed theta-power differences between conditions into two different sources: (1) differences in rhythm duration and (2) differences in power amplitudes during rhythmic events.

We first compared rhythm abundance using two-dimensional cluster-based permutation statistics (channels x frequency) in the a priori defined frequency range of 2-8Hz. In line with our findings in the main manuscript, involuntarily remembered full-hits compared to correct rejections showed higher theta abundance in midfrontal electrodes and in a frequency range of 4-6Hz (t_{sum} = 54.48; p_{corr} = .022; fig S7). During voluntary retrieval, full-hits induced higher theta abundance compared to correct rejections in two clusters. The first cluster was located in right frontal electrodes within a frequency range of 3.5-5 Hz (t_{sum} = 59.43; p_{corr} = .017), while the second cluster was located in midparietal electrodes with a frequency range of 2.5-4.5 Hz (t_{sum} = 59.61; p_{corr} = .017; fig S7). Although these clusters were smaller than in the original analysis, they largely overlap with their spatial distribution underlining that our results can be explained by theta oscillations.

Next, we compared power estimates during rhythmic events using cluster-based permutation statistics (channels x frequency x time) in the a priori defined frequency range of 2-8Hz. We did not observe any significant differences in theta-power between full-hits vs correct rejections during

involuntary or voluntary retrieval. Thus, differences in theta frequency bands can be primarily described by the duration or abundance of occurring theta oscillations.

Having established that theta-power differences between full-hits and correct rejections during both retrieval phases reflect differences in the abundance of theta oscillations, we finally aimed to test whether aperiodic slopes differ between full-hits and correct rejections. We therefore compared aperiodic slopes that were estimated using the eBOSC method (8). We averaged aperiodic slopes of each trial in the according full-hits and correct rejection conditions and entered them to a one-dimensional cluster-based permutation statistic (channels). During involuntary retrieval, slopes were steeper during full-hits compared to correct rejections in a cluster including midfrontal electrodes similar to the previously described theta abundance cluster (t_{sum} = -65.90; p_{corr} = .004; fig S7). Notably, this cluster additionally included left posterior electrodes that did not show differences in theta oscillations during involuntary retrieval in abundance or general theta-power effects in the 2-8Hz range. Similarly, during voluntary retrieval, full-hits were associated with steeper slopes than correct rejections (t_{sum} = -18.64; p_{corr} = .032; fig S7). Yet, this cluster consisted of electrodes in left frontal areas that did not overlap with theta abundance or theta-power effects of voluntary retrieval in the main analysis. Our analyses thus underline that involuntary and voluntary memory retrieval was associated with both: prolonged theta oscillations and steeper aperiodic slopes.
