# Supplementary material for: Involuntary and voluntary memory retrieval relies on distinct neural representations and oscillatory processes
Source: PLoS Biol. 2025 Aug 19;23(8):e3003258. doi: 10.1371/journal.pbio.3003258 (PMC12364361; doi:10.1371/journal.pbio.3003258)
Supplement: S9 Text — (PDF) [file pbio.3003258.s017.pdf]

S9 Text. Analyzing alpha/beta power decreases in contralateral electrodes during encoding

Previous studies showed alpha/beta power decreases during encoding of items in the left or right visual hemifield over contralateral electrodes (9, 10). Interestingly, these contralateral alpha/beta power decreases reoccurred during memory retrieval even if memory cues were presented in the center of the screen which was interpreted as the reactivation of sensory memory representations. To test this effect in our study, we first compared time-frequency data during encoding of items in the left vs right visual hemifield using cluster-based permutation tests during the time window of 0-1,500ms after item onset. As a result of contralateral alpha/beta power decreases, we expected a negative effect in the right hemisphere and a positive effect in the left hemisphere. However, we did not find a negative (T_{sum} = -3748.16, *p*s > .123) nor a positive cluster (T_{sum} = 212.41, *p*s > .639). For illustration, we show mean t-values during the time window 0.2-0.7 s in frequency bands of 8-20 Hz (see fig. S8) which has previously been suggested to be a prominent time window of contralateral visual processing (10). A potential reason for diverging findings from previous studies may be that simultaneous presentation of a central cue together with the lateralized item has reduced lateralized alpha/beta decreases. This may have led to more subtle and specific contralateral processing patterns of visual information which were identifiable using multivariate but not univariate approaches. As we did not find any contralateral alpha/beta power decreases during encoding, we decided to use multivariate encoding-retrieval similarity approaches to track sensory feature reactivation.
