# Supplementary material for: Involuntary and voluntary memory retrieval relies on distinct neural representations and oscillatory processes
Source: PLoS Biol. 2025 Aug 19;23(8):e3003258. doi: 10.1371/journal.pbio.3003258 (PMC12364361; doi:10.1371/journal.pbio.3003258)
Supplement: S1 Table — (DOCX) [file pbio.3003258.s018.docx]

| *Effect* | num  *df* | den  *df* | *F* | *p* |
| --- | --- | --- | --- | --- |
| Involuntary memories: RT source memory task | | | | |
| Low-level sensory reactivation | 1 | 459 | 0.57 | .453 |
| Involuntary memories: RT item memory task | | | | |
| Low-level sensory reactivation | 1 | 459 | 0.19 | .663 |
| Voluntary memories: RT source memory task | | | | |
| Item-specific reactivation | 1 | 295 | 0.58 | .447 |
| Voluntary memories: RT item memory task | | | | |
| Item-specific reactivation | 1 | 319 | 0.05 | .826 |
|  |  |  |  |  |
